# Synergistic effects of exosomal crocin or curcumin compounds and HPV L1-E7 polypeptide vaccine construct on tumor eradication in C57BL/6 mouse model

**DOI:** 10.1371/journal.pone.0258599

**Published:** 2021-10-14

**Authors:** Elnaz Abbasifarid, Azam Bolhassani, Shiva Irani, Fattah Sotoodehnejadnematalahi

**Affiliations:** 1 Department of Biology, School of Basic Sciences, Science and Research Branch, Islamic Azad University, Tehran, Iran; 2 Department of Hepatitis and AIDS, Pasteur Institute of Iran, Tehran, Iran; Lobachevsky University, RUSSIAN FEDERATION

## Abstract

Cervical cancer is the most common malignant tumor in females worldwide. Human papillomavirus (HPV) infection is associated with the occurrence of cervical cancer. Thus, developing an effective and low-cost vaccine against HPV infection, especially in developing countries is an important issue. In this study, a novel HPV L1-E7 fusion multiepitope construct designed by immunoinformatics tools was expressed in bacterial system. HEK-293T cells-derived exosomes were generated and characterized to use as a carrier for crocin and curcumin compounds. The exosomes loaded with crocin and curcumin compounds as a chemotherapeutic agent (ExoCrocin and ExoCurcumin) were used along with the L1-E7 polypeptide for evaluation of immunological and anti-tumor effects in C57BL/6 mouse model. *In vitro* studies showed that ExoCrocin and ExoCurcumin were not cytotoxic at a certain dose, and they could enter tumor cells. *In vivo* studies indicated that combination of the L1-E7 polypeptide with ExoCrocin or ExoCurcumin could produce a significant level of immunity directed toward Th1 response and CTL activity. These regimens showed the protective and therapeutic effects against tumor cells (the percentage of tumor-free mice: ~100%). In addition, both ExoCrocin and ExoCurcumin represented similar immunological and anti-tumor effects. Generally, the use of exosomal crocin or curcumin forms along with the L1-E7 polypeptide could significantly induce T-cell immune responses and eradicate tumor cells.

## Introduction

Cervical cancer is the fourth most common cancer in women, ranking after breast cancer, colorectal cancer and lung cancer, respectively [1]. Cervical cancer is caused by persistent infection with a high-risk type of human papillomavirus (hrHPV; 84% of all HPV-related cancers worldwide; [2]). The World Health Organization (WHO) recommended HPV vaccines as a main strategy to suppress HPV-related diseases especially cervical cancer (WHO, 2016). Three prophylactic HPV vaccines have been licensed based on non-infectious virus-like particles (VLPs). However, the coverage of HPV types was limited in these vaccines, and their use in pregnant women was not advised. Therefore, development of vaccines with broader preventive and therapeutic effects and better safety profile is recommended [3]. The current standard of care for cervical cancer is chemo-radiation therapy (CRT), however it has a major negative effect on the quality of life for patients and sometimes with a low survival rate. Hence, there is an urgent need to develop an effective and easy therapy against cervical cancer in the world [4].

HPV genome contains early regions encoding regulatory proteins (E1, E2, E4, E5, E6, E7 and E8), late regions encoding capsid proteins (L1 and L2), and a non-coding region (or long control region) [5]. HPV E6 and E7 proteins are necessary for tumor progression suggesting them as ideal foreign antigen targets for development of therapeutic vaccines [4]. The studies indicated that cytotoxic T lymphocytes (CTL) are likely the most effective immunological effector mechanism [4, 6]. The studies showed that a thioredoxin-based L2-E7 nanoparticle vaccine [7], Manganese-doped silica-based nanoparticle vaccine [8], and particle-forming liposomes [9] were used for treating the established murine HPV E7-expressing solid TC-1 tumors.

Since ancient times, plants have been used to treat different types of lesions with promising results. More than 60% of anticancer drugs were derived from natural compounds [10]. Bioactive natural products play an important role in health maintenance as novel therapeutic agents. The studies showed that two major compounds including carotenoids and retinoids have several similar biological activities such as antioxidant properties, inhibition of tumor growth, and induction of apoptosis [11]. For example, crocin (the digentiobiosyl ester of crocetin-α-crocin; a carotenoid chemical compound) and curcumin (a yellow substance belonging to the polyphenols superfamily) are one of the most important ingredients of saffron (*Crocus sativus* L.) and turmeric, respectively which have anti-tumor/ anti-metastatic/ anti-angiogenic, anti-oxidant and chemopreventive properties *in vitro*/ *in vivo* [12–14].

Crocin treatment showed a cardioprotective effect in doxorubicin-treated rats. The anti-inflammatory properties of crocin included the reduced levels of COX-2 and TNF-*α* mRNA, and low iNOS expression and nitric oxide production through down-regulation of NF-*κ*B activity [15]. Crocin inhibits tumor growth in a variety of cancers indicating its potential use as a cancer chemotherapeutic and also preventive agent. Crocin could induce apoptosis in tumor cells by down-regulating the expression of Bcl-2, survivin, cyclin D1, and up-regulating the expression of Bax in BALB/c xenograft tumor. On the other hand, it was found that crocin interacts with tubulin and increases the polymerization of microtubules *in vitro* [16]. Curcumin inhibits the STAT3 and NF-ĸB signaling pathways, which play key roles in cancer progression. Also, curcumin may act by suppressing the Sp-1 activation and its downstream genes including ADEM10, calmodulin, EPHB2, HDAC4, and SEPP1 in a dose-dependent manner in colorectal, bladder and lung cancer cells [17].

The studies indicated that poor absorption, quick metabolism, and rapid systemic elimination led to the restriction of crocin and curcumin bioavailability. Thus, it is required finding effective methods of delivery [18, 19]. Exosomes are small endosome-derived vesicles (50–100 nm in size) containing nucleic acid and protein cargos which are secreted by all types of cells *in vitro*, and also found naturally in body fluids [20, 21]. Several reports have already demonstrated their potential for therapeutic drug delivery in various animal models. The majority of these studies have been focused on cancer therapy [22–25]. Due to their small size, they may achieve passive targeting to tumors via the enhanced permeation and retention (EPR) effect [26].

**Fig 1:**
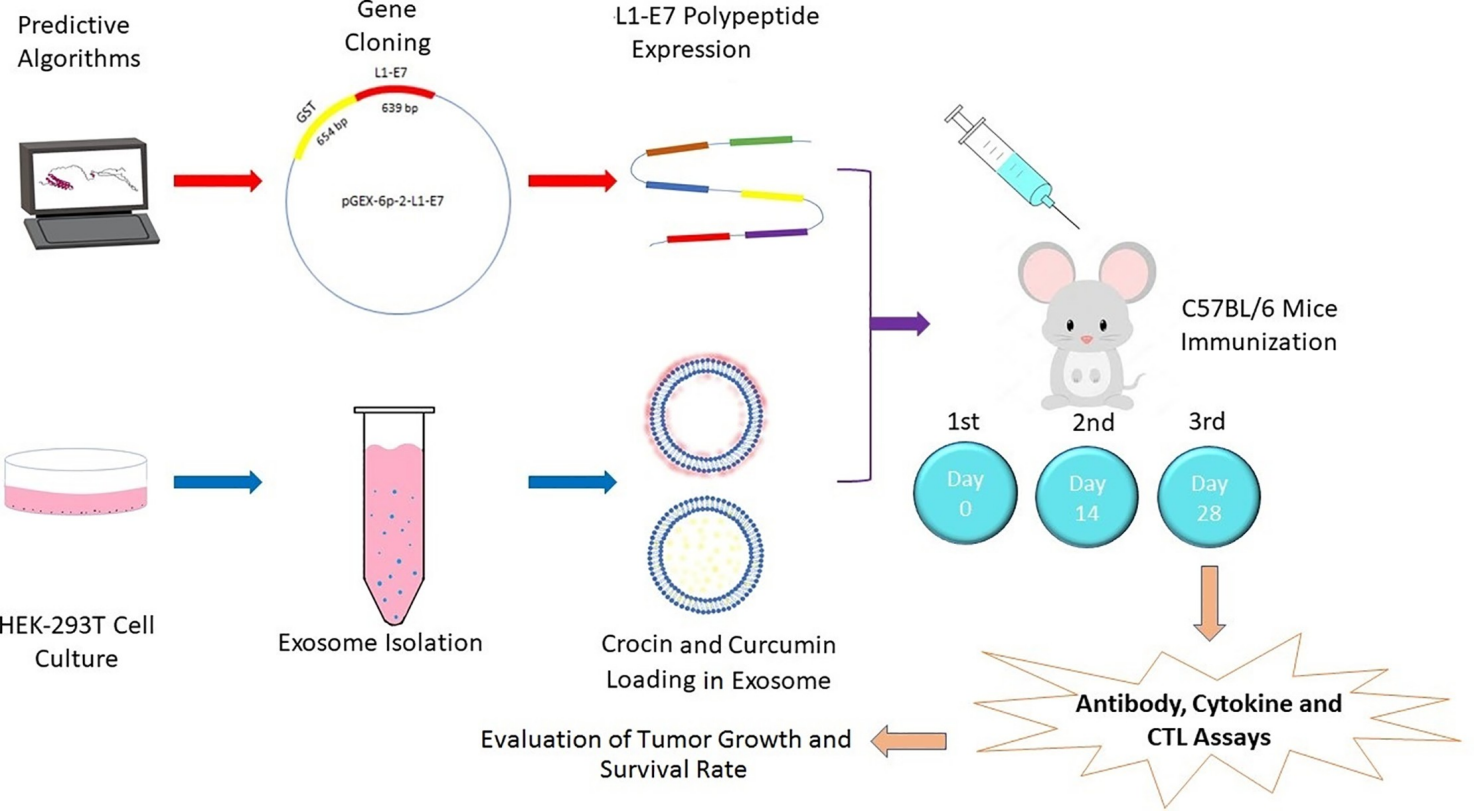
Schematic overview of different approaches in this study.

In this study, combination of immunotherapy (a polypeptide construct) with chemotherapy (Exosomal crocin or curcumin forms) was used to evaluate immune responses, and eradicate TC-1 tumors. The polypeptide construct was designed based on HPV L1 and E7 proteins using *in silico* studies. Moreover, the HEK-293T-derived exosomes were used to deliver crocin and curcumin compounds *in vitro* and *in vivo*. **Fig 1** shows the schematic model of this study as a brief.

## Materials and methods

### Preparation of a novel multiepitope construct

**Fig 2:**
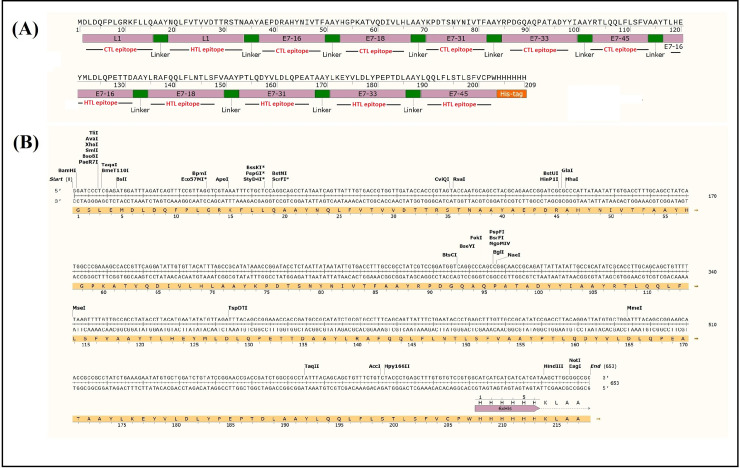
The L1-E7 multiepitope peptide and DNA constructs. A) The L1-E7 multiepitope peptide construct with 6xHis-tag: The helper T lymphocyte (HTL) and cytotoxic T lymphocyte (CTL) epitopes were shown as underline text; B) The multiepitope DNA construct with the restriction enzyme sites.

In our previous study [27], twelve T-cell epitopes derived from HPV L1 and E7 proteins were selected using various *in silico* predictions such as human MHC class I/ II epitope prediction (using NetMHCpan 4.0 server/ NetMHCII 2.3 server), MHC class I processing prediction (based on the immune epitope database or IEDB recommendations), conservancy analysis (using the IEDB conservancy tool), population coverage (using the IEDB population coverage tool), prediction of cytokine induction (*e*.*g*., IL-4 and IFN-γ using IL-4pred and IFNpred tools), prediction of allergenicity (using AllerTOP v2.0 and AllergenFP servers), toxicity (using ToxinPred web server), immunogenicity (using IEDB immunogenicity predictor tool), antigenicity (using ANTIGENpro and VaxiJen v2.0 servers), hemolytic potency (using hemolytic web server), cross reactivity (using peptide matching server), and peptide-MHC molecular docking (using the GalaxyPepDock web server). The L1 epitopes were selected from two low-risk (6 & 11) and sixteen high-risk (16, 18, 31, 33, 35, 39, 45, 51, 52, 55, 58, 59, 68, 73, 82 & 83) HPV strains, and the E7 epitopes were selected from five high-risk strains (16, 18, 31, 33 & 45) with the highest impact on cervical cancer. Afterwards, a novel multiepitope construct using twelve selected epitopes and AAY linker was designed (**Fig 2A**). For expression of the L1-E7 polypeptide in the next steps, different cutting sites were considered in its DNA construct (**Fig 2B**). The potential B-cell epitopes were then determined in the full-length of multiepitope construct by Bepipred 2.0. Next, to identify the physicochemical properties and protein solubility of the designed construct, ProtParam, and Protein-Sol web servers were applied. After that, the secondary and tertiary structures were predicted by RaptorX, PSIPREDm and I-TASSER servers, respectively. Moreover, refinement of 3D structure was done by ModRefiner, and Galaxy Refine. Finally, 3D structure validation was performed using ERRAT, ProSA-web and RAMPAGE servers [27].

### Prediction of the binding affinity of CTL epitopes to mouse MHC alleles

In this study, the binding affinity of the selected epitopes to mouse MHC alleles (both class I and class II) was predicted, as well. To predict the binding affinity of CTL epitopes to mouse MHC class I alleles, three web servers such as IEDB MHC-1 prediction tool (http://tools.iedb.org/mhci/), NetMHCpan4.1 (http://www.cbs.dtu.dk/services/NetMHCpan/), and SYFPEITHI (http://www.syfpeithi.de/bin/MHCServer.dll/EpitopePrediction.htm) were utilized. Default threshold settings were used in prediction tools.

### Prediction of the binding affinity of HTL epitopes to mouse MHC alleles

Helper T-lymphocyte (HTL) immune responses help in pathogen clearance. Herein, to predict the binding affinity of HTL epitopes to mouse MHC class II alleles, IEDB MHC-II prediction tool (http://tools.iedb.org/mhcii/), and SYFPEITHI (http://www.syfpeithi.de/bin/MHCServer.dll/EpitopePrediction.htm) were applied. Default threshold settings were used in prediction tools.

### Analysis of peptide-MHC docking

The binding of the selected epitopes to human MHC was previously investigated [27]. Herein, their binding to mouse MHC was evaluated, as well. The GalaxyPepDock peptide-protein flexible docking web server (http://galaxy.seoklab.org/cgibin/submit.cgi?type=PEPDOCK) was used to evaluate the formation of peptide-mouse MHC class I complex. The PDB files of mouse MHC alleles were extracted from RCSB (https://www.rcsb.org).

### Molecular docking of the L1-E7 multiepitope construct with various toll-like receptors

Toll-like receptors (TLRs) have critical roles in the stimulation of host antimicrobial defenses [28]. Molecular docking between the multiepitope vaccine construct and major TLRs was performed to predict possible binding orientation of the construct. The PDB files of TLRs (TLR-2 PDB ID: 2Z7X, TLR-3 PDB ID: 1ZIW, TLR-4 PDB ID: 3FXI, TLR-5 PDB ID: 3J0A, TLR-8 PDB ID: 3W3G, and TLR-9 PDB ID: 3WPB) were extracted from RCSB (https://www.rcsb.org). Then, ClusPro 2.0 web server (https://cluspro.bu.edu/; [29]) was utilized to perform the TLRs-multiepitope construct binding interactions.

### Generation of the recombinant L1-E7 polypeptide

The L1-E7 multiepitope DNA construct was synthesized in pUC57 cloning vector, and subsequently was subcloned in the *Bam*HI/*Not*I cloning sites of pGEX-6p2 expression vector. The Glutathione S-transferase (GST)-L1-E7 expression was performed in *E*. *coli* strains BL21 and Rosetta under different conditions including IPTG concentrations (rang of 0.1–1 mM), temperatures (30°C and 37°C), OD_600_ (rang of 0.5–1) and times (1, 2, 3, 4 and 16 h). After confirming protein expression by sodium dodecyl sulphate-polyacrylamide gel electrophoresis (SDS-PAGE), the GST-L1-E7 protein was purified by Ni-NTA beads (Qiagen) under denaturing conditions using constant concentration of urea and pH gradient (from 8 to 4.5). Elution fractions containing GST-L1-E7 protein were pooled, dialyzed in phosphate-buffered saline 1X (PBS 1X) for removing urea and other salts, and confirmed by SDS-PAGE and western blot analysis using anti-His tag antibody (1: 10000 *v/v*, Abcam). Then, PreScission protease was utilized to remove GST-tag from the purified protein. Finally, the purified protein was dialyzed, and its concentration was measured by Bradford protein assay kit and NanoDrop spectrophotometer. On the other hand, the LAL assay (QCL-1000) was done to determine the contamination with bacterial endotoxin which was less than 0.4 EU/mg.

### Isolation of HEK-293T cell-derived exosomes

HEK-293T cells were cultured in 25 cm^2^ flasks containing RPMI 1640 medium supplemented with 10% exosome-depleted bovine serum (FBS; System Bioscience) and 1% Pen-Strep antibiotic (Sigma). The cells were incubated at 37°C in 5% CO_2_ for 48 h. Then, 24 h after cell passage (the cell confluency was 70–80%), the culture medium was slowly collected. Finally, HEK-293T-derived exosomes were isolated using ExoQuick-TC kit (System Biosciences). According to ExoQuick-TC protocol, the collected medium was centrifuged at 3000 g for 15 min to remove cells and debris. In the next step, 1 ml of ExoQuick-TC buffer was mixed with 5 ml of culture medium, and incubated at 4°C for 24 h. The mixture was centrifuged at 3000 g for 10 min to precipitate exosomes. The Bradford test was performed to measure protein levels at this stage. After that, the pellet was resuspended in various buffers (based on kit), and finally, the solution containing pure exosome was obtained. The BCA protein assay kit (Thermo Fisher Scientific) was used to measure protein content at this stage.

### Confirmation of exosomes

Scanning electron microscope (FEI Nova Nano SEM 450), and western blotting were used to detect the presence of the isolated exosomes, and confirm their nature, respectively. For western blotting, 60 μl of the isolated exosomes were solubilized in 30 μl of RIPA buffer (DNAbiotech), and protein concentration was quantified by Bradford assay. HEK-293T cells (negative control) and exosome proteins (about 30 μg of total protein) were separated using 10% SDS polyacrylamide gel. Protein bands were transferred onto nitrocellulose membrane (Bio-Rad). After incubating in BSA blocking buffer (TBS 1X, 0.1% tween 20, 2.5% BSA) for 2 h, the samples were incubated with anti-CD63 antibody (Abcam; 1:1000) and also anti-CD9 antibody (Abcam; 1:1000) for 24 h in 4°C. Then, the membrane was incubated with the mouse total anti-IgG HRP-conjugated secondary antibody (1:10000) for 1 h at room temperature. Finally, 3, 3′-Diaminobenzidine solution (DAB/ H_2_O_2)_ was used to detect the bands.

### Preparation of crocin and curcumin

The crocin and curcumin solutions were prepared by dissolving crocin powder in PBS and curcumin powder (Sigma) in ethanol for the next uses. For achieving high amounts of crocin, it was extracted by adsorption chromatography using aluminum oxide 90-active according to our previous studies [30, 31].

### ExoCrocin and ExoCurcumin preparation

Two methods were investigated for loading crocin and curcumin into exosomes including sonication and freeze-thaw cycles. The crocin solution in PBS 1X (2 mM) and the curcumin solution in ethanol/PBS (2 mM) were mixed with exosomes in the fixed proportion (1: 4) [32, 33]. The final solutions were sonicated (duty 80%, time 36s, and power 10%) and cooled down on the ice for 2 min. This process was done twice. For the freeze-thaw method, crocin or curcumin solutions were added to exosomes, and incubated for 30 min. Then, the mixture was rapidly frozen at -75°C, and thawed at 37°C. The freeze-thaw cycle was repeated three times [34]. Non-entrapped crocin and curcumin were separated by centrifugation at 14,000 rpm for 30 min. For determination of crocin and curcumin concentration in the isolated exosomes, exosomal crocin (ExoCrocin) and exosomal curcumin (ExoCurcumin) were dissolved in RIPA buffer. The optical density was measured at 430–450 nm for ExoCrocin and at 410–430 nm for ExoCurcumin after dissolving in RIPA buffer [35]. The purity and integrity of exosomes before and after drug (crocin or curcumin) loading were analyzed using LEO 906 Transmission Electron Microscope (TEM; Zeiss).

### Fluorescence imaging

Fluorescence imaging was performed to detect the entry of ExoCrocin and ExoCurcumin into TC-1 cell lines. Briefly, the cancerous TC-1 cells (5% RPMI) were seeded in 12-well plate (10000 cells/ well), and exposed to crocin, ExoCrocin, curcumin and ExoCurcumin. The plate was incubated for 24 h at 37°C in humidified incubator with 5% CO_2_ atmosphere. After 24 h, the cells were studied using a fluorescence microscope (INV-100; BEL ENGINEERING).

### MTT assay

The viability of the non-cancerous HEK-293T was studied after seeding 10000 cells/ well in a 96-well plate, and then incubating with fresh RPMI medium containing the empty exosome (200μg), crocin (2 mM), ExoCrocin (2 mM crocin in 200μg exosome), curcumin (2 mM), ExoCurcumin (2 mM curcumin in 200μg exosome), and the L1-E7 polypeptide (5 μg) for 48 h. After incubation, 3-(4, 5-dimethyl thiazol-2-yl)-2, 5-diphenyl tetrazolium bromide (MTT; final concentration: 0.5 mg/ml) was added to each well. The cells were incubated at 37°C in a humidified 5% CO_2_ for 3h and next, the insoluble formazan (purple) was dissolved in dimethyl sulfoxide (DMSO). Finally, absorption (optical density) was measured at 570 nm by an ELISA reader. The non-treated cells were used as a negative control. The cell viability was calculated from the following equation:

$$Cell\;viability\;(\%)=mean\;OD_{test}‐mean\;OD_{control}/mean\;OD_{control}$$

### Mice immunization

Inbred C57BL/6 female mice, 5–7 week old, were obtained from the breeding stocks maintained at Pasteur Institute of Iran under specific pathogen-free conditions. All procedures were performed according to approved protocols and in accordance with recommendations for the proper use and care of laboratory animals which were evaluated by Islamic Azad University-Science and Research Branch (Approval ID: IR.IAU.SRB.REC.1398.181; Approval Date: 2020-02-22). Eight groups of female C57BL/6 mice (n = 8 per group) were injected on days 0, 14 and 28 with the L1-E7 polypeptide (5 μg) + Montanide 720 adjuvant (the polypeptide: adjuvant ratio is 30: 70 *v/v*, G1), the L1-E7 polypeptide (5 μg) + ExoCrocin (2 mM crocin loaded in 200μg exosome; G2), ExoCrocin (2 mM crocin; G3), the L1-E7 polypeptide (5 μg) + ExoCurcumin (2 mM curcumin loaded in 200μg exosome; G7), ExoCurcumin (2 mM curcumin loaded in 200μg exosome; G8). Groups 4–6 were injected with PBS, Montanide and the empty exosome as control groups, respectively (**Table 1**). The L1-E7 polypeptide was injected subcutaneously (*s*.*c*.), and the empty exosome, ExoCurcumin and ExoCrocin were administered Intraperitoneally (*i*.*p*.) in mice.

**Table 1:** Mice immunization for protective study.

Group	1^st^ injection (Day 0)	2^nd^ injection (Day 14)	3^rd^ injection (Day 28)	Challenge (Day 49)
**G1**	L1-E7 polypeptide + Montanide	L1-E7 polypeptide + Montanide	L1-E7 polypeptide + Montanide	TC-1
**G2**	L1-E7 polypeptide + Montanide +ExoCrocin	L1-E7 polypeptide + Montanide +ExoCrocin	L1-E7 polypeptide + Montanide +ExoCrocin	TC-1
**G3**	ExoCrocin	ExoCrocin	ExoCrocin	TC-1
**G4**	PBS	PBS	PBS	TC-1
**G5**	Montanide	Montanide	Montanide	TC-1
**G6**	Empty Exosome	Empty Exosome	Empty Exosome	TC-1
**G7**	L1-E7 polypeptide + Montanide +ExoCurcumin	L1-E7 polypeptide + Montanide +ExoCurcumin	L1-E7 polypeptide + Montanide +ExoCurcumin	TC-1
**G8**	ExoCurcumin	ExoCurcumin	ExoCurcumin	TC-1

### Monitoring tumor progression

For *in vivo* protection experiment, C57BL/6 mice were vaccinated three times with a two-week interval (**Table 1**). Then, vaccinated mice were subcutaneously challenged in the right flank with 0.1 × 10^6^ TC-1 tumor cells three weeks after the last immunization. Tumor growth and survival rates were monitored by palpation twice a week for 65 days. Tumor volume was calculated using the formula: V = (a^2^b)/2. Mice were euthanized when tumor diameter exceeded >5% of body weight.

### Antibody assay

Three weeks after the last immunization, sera were collected from whole blood samples of each group. The levels of total IgG, IgG1, IgG2a and IgG2b were measured in the sera with goat anti-mouse immunoglobulin antibodies (diluted 1:10,000 in 1% BSA/PBS-Tween, Sigma) by indirect ELISA. The coated antigen was the recombinant L1-E7 polypeptide. 3, 3′, 5, 5′-tetramethylbenzidine (TMB) was used as substrate.

### Cytokine assay

To determine the secretion of IFN-γ and IL-4 cytokines, three mice from each group were chosen three weeks after the last injection. Mice were sacrificed following ketamine/xylazine anesthesia and the spleens were removed under sterile conditions. The red blood cell-depleted pooled splenocytes (2 × 10^6^ cells/ml) were seeded in 48-well plates exposed to the recombinant L1-E7 polypeptide (5 μg). Also, RPMI 5% and concanavalin A (Con A; 5 μg) were considered as negative and positive controls, respectively. The supernatants were harvested to assess the secretion of IFN-γ and IL-4 by a sandwich-based ELISA system (Mabtech, Sweden) as stated by the manufacturer’s instructions. All data were reported as mean ± SD for each set of samples. The lymphocyte proliferation was assessed by MTT and indicated as stimulation index (SI).

### Evaluation of granzyme B

The P815 target cells (T) were seeded into 96-well plates (2 × 10^4^ cells/ well) and incubated with the L1-E7 polypeptide (~ 30 μg/ ml) for 24 h. Then, the prepared splenocytes (Effector cells: E) were added to the target cells at an E: T ratio of 100:1, and incubated for 6 h. Finally, the supernatants were harvested to measure the Granzyme B concentration by ELISA (eBioscience kit) according to the manufacturer’s instruction.

### Therapeutic effects

For therapeutic tests of the established TC-1 tumors, three groups of four female C57BL/6 mice were considered. Briefly, four mice in each group were subcutaneously injected with 0.1 × 10^6^ TC-1 tumor cells in right flank, and then one week after tumor challenge, mice received various regimens three times with a two-week interval (**Table 2**). Finally, tumor growth was detected two times a week for 65 days. The L1-E7 polypeptide was injected subcutaneously (*s*.*c*.), and ExoCurcumin and ExoCrocin were administered intratumorally (*i*.*t*.) in mice.

**Table 2:** Mice immunization for therapeutic effects.

Groups	Challenge (Day 0)	1^st^ injection (Day 7)	2^nd^ injection (Day 21)	3^rd^ injection (Day 35)
**G1**	TC-1	L1-E7 polypeptide + Montanide + ExoCrocin	L1-E7 polypeptide + Montanide + ExoCrocin	L1-E7 polypeptide + Montanide + ExoCrocin
**G2**	TC-1	L1-E7 polypeptide + Montanide + ExoCurcumin	L1-E7 polypeptide + Montanide + ExoCurcumin	L1-E7 polypeptide + Montanide + ExoCurcumin
**G3**	TC-1	PBS	PBS	PBS

### Statistical analysis

Statistical analyses were performed by Prism software (GraphPad, USA) to determine the differences between the control and test groups using one-way ANOVA and student’s *t*-test. Survival rate (*i*.*e*., the percentage of tumor-free mice) was evaluated using the log-rank (Mantel-Cox) test. The value of *p* < 0.05 was statistically considered significant. The results were determined from two-independent experiments.

## Results

### Preparation of the multiepitope construct

The L1-E7 multiepitope construct was composed of the ^458^DLDQFPLGRKFLLQ^471^ (L1), ^327^NQLFVTVVDTTRSTN^341^ (L1), ^45^AEPDRAHYNIVTF^57^ (HPV16 E7), ^2^HGPKATVQDIVLHL^15^ (HPV18 E7), ^46^KPDTSNYNIVTF^51^ (HPV31 E7), ^40^RPDGQAQPATADYYI^54^ (HPV33 E7), ^85^RTLQQLFLSFV^95^ (HPV45 E7), ^7^TLHEYMLDLQPETTD^21^ (HPV16 E7), ^83^LRAFQQLFLNTLSFV^97^ (HPV18 E7), ^6^PTLQDYVLDLQPEAT^20^ (HPV31 E7), ^8^LKEYVLDLYPEPTDL^22^ (HPV33 E7), and ^87^LQQLFLSTLSFVCPW^101^ (HPV45 E7) epitopes, respectively as shown in **S1 Table**. The AAY linker was located between epitopes to improve the proteasomal processing of the construct.

Herein, these selected CTL and HTL epitopes bound to human MHC alleles were studied to bind mouse MHC alleles using various web servers (**S2 & S3 Tables**).

The peptide-MHC interaction between mice MHC class I alleles and the selected epitopes was investigated using GalaxyPepDock web server. The highest interaction similarity scores were illustrated in **S4 Table**.

Docking scores between MHC alleles and peptides were calculated by global docking algorithms. At first, the MHC-I structure data were retrieved from the RCSB PDB server. Then, the potential epitopes and MHC PDB data were independently submitted to the server. The highest interaction similarity scores were obtained from the GalexyPepDock server. Higher rate shows better quality of peptide-MHC interactions. The mean interaction similarity scores for the H2-Db and H2-Kb alleles in C57BL/6 mouse indicated that they had high interaction similarity scores as previously reported in human [27]. Moreover, some examples of peptide-MHC interactions between mice MHC class I alleles and CTL epitopes were shown in **S1 Fig**. Subsequently, total 30 models were generated for each molecular docking between the L1-E7 multiepitope construct and TLR-2, TLR-3, TLR-4, TLR-5, TLR-8, and TLR-9 receptors using ClusPro 2.0. The interaction models with the lowest energy scores were selected as shown in **S2 Fig**. The lowest energy levels achieved for TLR-2/ TLR-3/ TLR-4/ TLR-5/ TLR-8/ TLR-9-multiepitope construct interaction were -998, -1101.9, -1112.5, -1484, -1148.5, and -1114.6, respectively indicating the highest binding affinity among the docked complexes.

Finally, the L1-E7 multiepitope construct along with an amino-terminal 6xHis-tag was synthesized as a synthetic DNA construct in pUC57 vector (the length of L1-E7 gene was ~ 645 bp; **Fig 2**).

### Generation of the recombinant L1-E7 polypeptide

The GST-L1-E7 protein was expressed in *E*. *coli* BL21 strain, IPTG concentration of 1mM, optical density of 0.6–0.7, and time incubation of 3h after induction. The SDS-PAGE analysis showed a clear band of ~ 51 kDa for GST-L1-E7 fusion protein (**S3A Fig**). Furthermore, the recombinant GST-L1-E7 protein was detectable using anti-His antibody in western blotting (**S3B Fig**). The protein purification was performed by affinity chromatography under denaturing method. On the other hand, SDS-PAGE analysis indicated that the L1-E7 polypeptide (~24 kDa) was successfully separated from GST-tag (~27 kDa) after incubation with PreScission protease. The concentration of L1-E7 polypeptide was obtained approximately 2.73 mg/ml.

### Generation of ExoCrocin and ExoCurcumin

**Fig 3:**
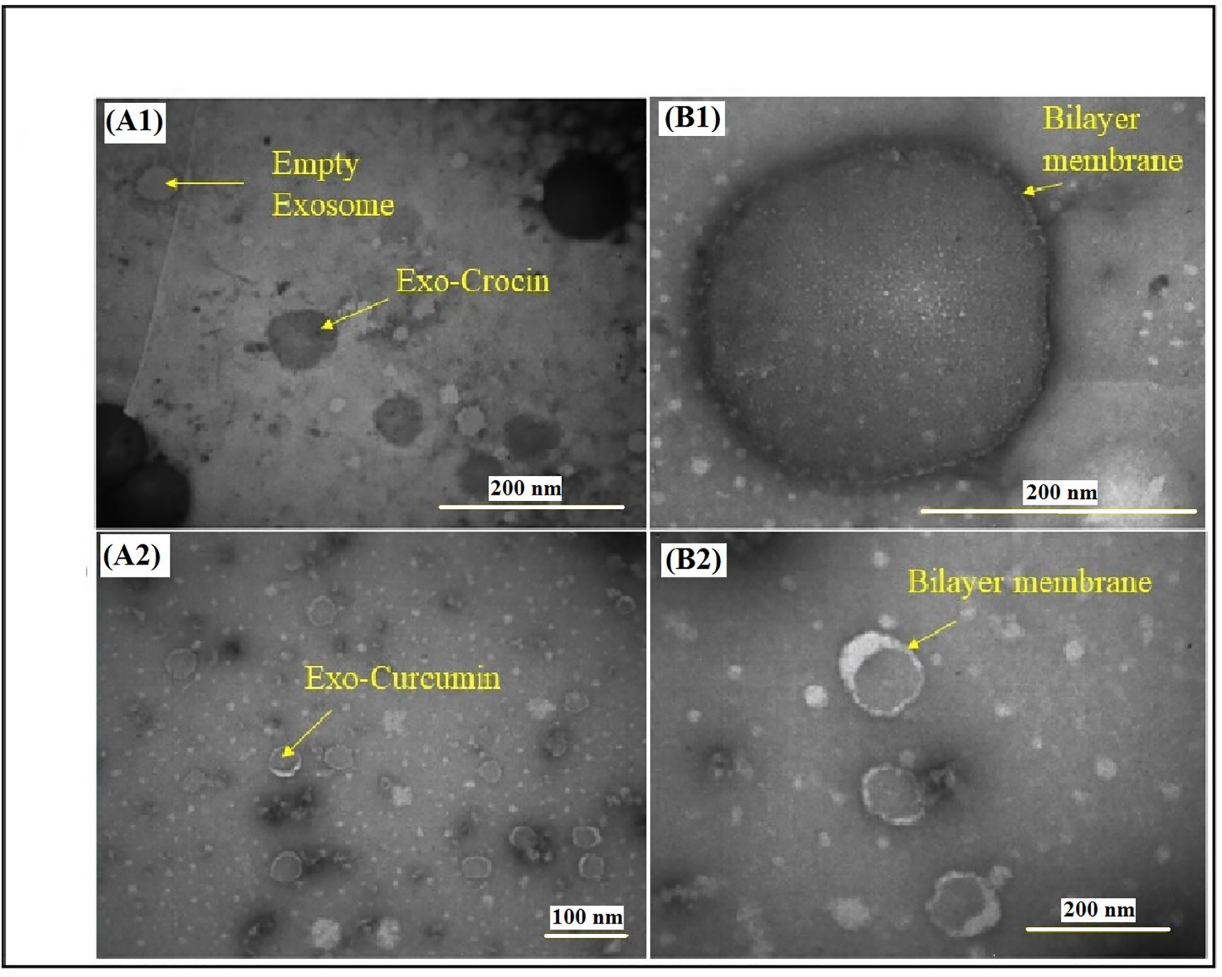
Characterization of ExoCrocin and ExoCurcumin using TEM images: An increased size, and color change to dark gray could be observed for ExoCrocin (A1); The inside of the exosomes is dark after curcumin loading, but no significant change in size (A2); Lipid membrane and integrity of exosomes after crocin (B1) and curcumin (B2) loading is clearly visible.

HEK-293T cell-derived exosomes were prepared and quantified by Bradford assay based on ExoQuick protocol. Approximately 2.8–3.5 mg protein equivalent of exosomes per column was obtained in purification step indicating sufficient amount of exosomes. The results of scanning electron microscopy (SEM) showed that the size of the exosomes was between 30 and 100 nm (**S4A Fig**). The exosomes’ shape and membrane integrity were confirmed using a transmission electron microscopy (TEM; **S4B Fig**). The exosome identification was confirmed by anti-CD63 and anti-CD9 antibodies as the clear bands of ~ 45–100 kDa in western blotting. Two methods for crocin and curcumin incorporation into exosomes were used including sonication and freeze-thaw. The concentrations of crocin and curcumin loaded in exosomes by absorbance assay showed that freeze-thaw method is better than sonication under the used conditions (**S5 Table**). ExoCrocin and ExoCurcumin detected by TEM showed that after crocin loading in exosome, the exosomes became larger (about 150–250 nm) but curcumin-loaded exosomes had smaller sizes in comparison with ExoCrocin (**Fig 3A1 & 3A2**). Also, the membrane integrity of ExoCrocin and ExoCurcumin was proved as shown in **Fig 3B1 & 3B2**.

### Detection of ExoCrocin and ExoCurcumin delivered into TC-1 cells

**Fig 4:**
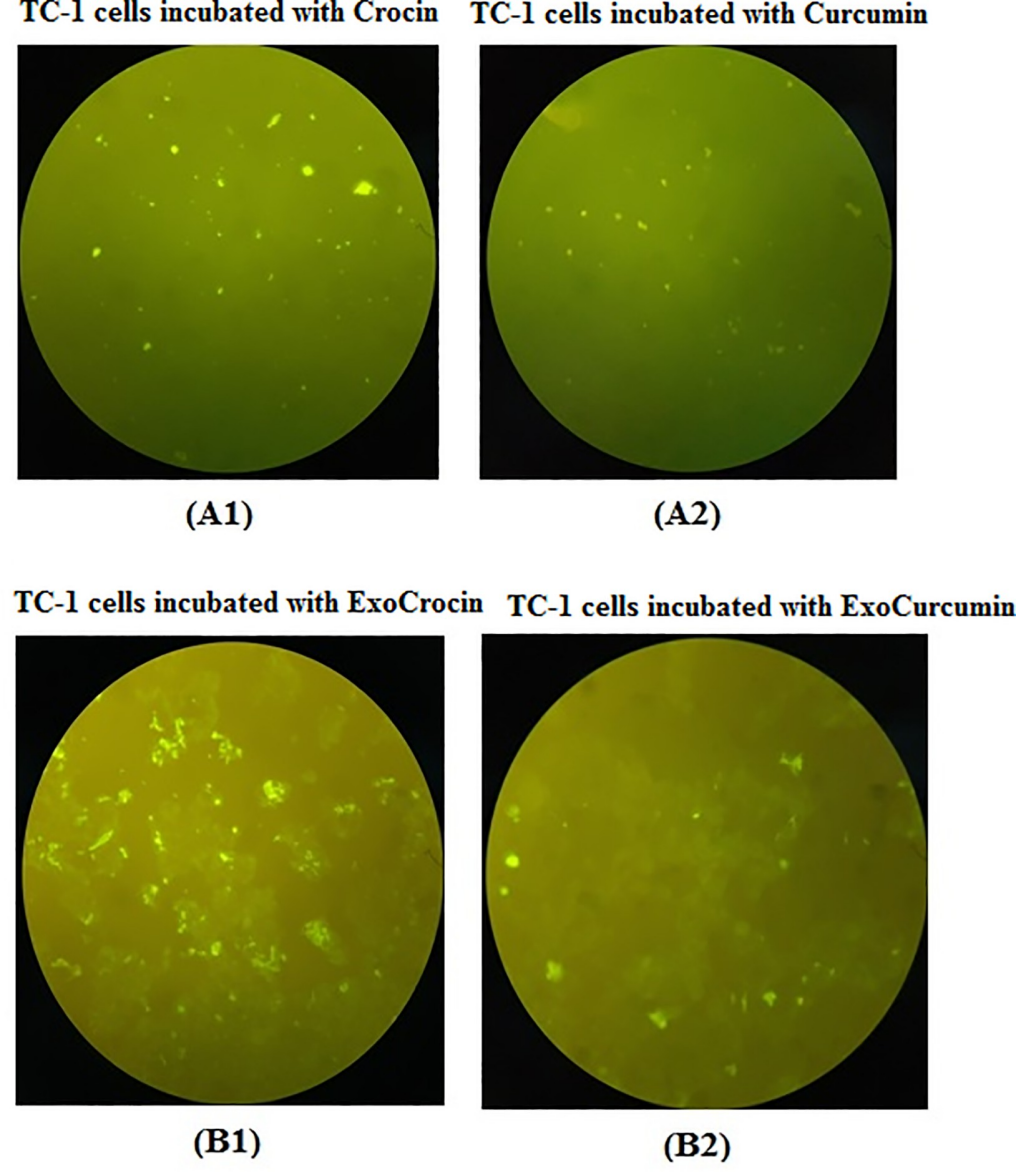
Delivery of exosomal crocin and curcumin forms in TC-1 cells using fluorescence microscopy: TC-1 cell line was incubated with crocin, curcumin (A1 & A2) and exosomal forms of these compounds (B1 & B2). (A1) TC-1 incubated with crocin, (A2) TC-1 incubated with curcumin, (B1) TC-1 incubated with ExoCrocin, and (B2) TC-1 incubated with ExoCurcumin.

The spectral properties of crocin and curcumin (*i*.*e*., an absorbance peak at 440 nm and 420 nm, respectively) were utilized in detecting the compounds under experimental conditions. Our fluorescence microscopy data showed that both ExoCrocin and ExoCurcumin (as detected in green color) have the ability to enter TC-1 cells. The content of crocin and curcumin was increased with exosome transmission within cells (**Fig 4**).

### MTT assay

MTT assay showed that the L1-E7 polypeptide and the empty exosome were safe, and did not have cytotoxic effects on non-cancerous HEK-293T cells (Viability percentage: 95–98%). The viability percentages for crocin, curcumin, ExoCrocin and ExoCurcumin were 78–81%, 76–79%, 90–92% and 91–94%, respectively. As observed, the exosomal forms of crocin and curcumin had no significant cytotoxic effects on HEK-293T cells at a certain concentration. The untreated cells had a viability percentage about 98–99%.

### Antibody assay

**Fig 5:**
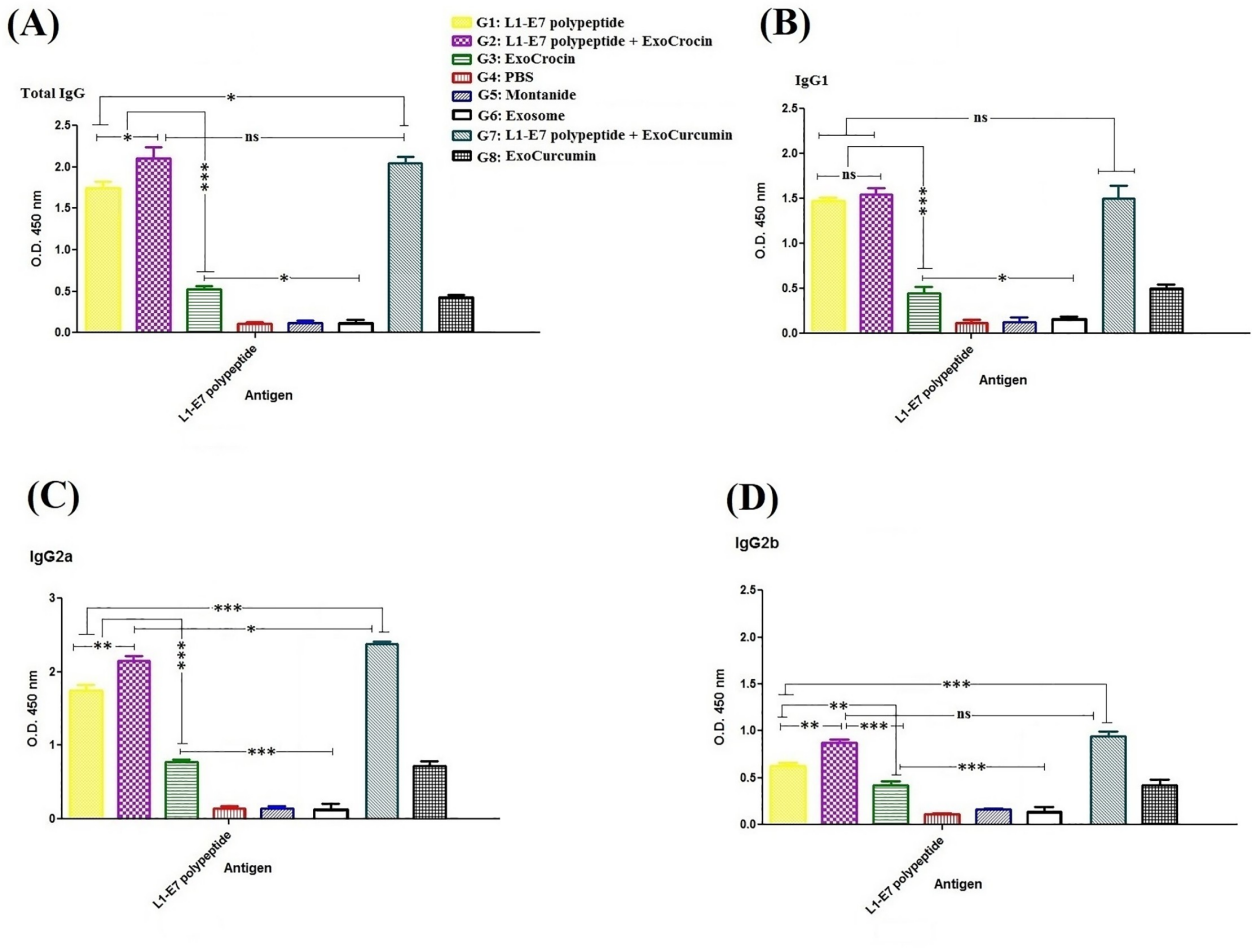
Evaluation of total IgG (A), IgG1 (B), IgG2a (C) and IgG2b (D) antibody responses in each group using indirect ELISA: All analyses were performed in duplicate for each sample. The results were shown as mean absorbance at 450 nm ± SD. ns: non-significant; * *p* < 0.05; **p < 0.01; *** *p* < 0.001.

The levels of L1-E7 polypeptide, L1-E7 polypeptide + ExoCrocin, ExoCrocin, L1-E7 polypeptide + ExoCurcumin and ExoCurcumin-specific total IgG, IgG1, IgG2a and IgG2b antibodies were measured in mice sera using ELISA (**Fig 5**). There was no significant difference in the level of IgG1 among L1-E7 polypeptide (G1), L1-E7 polypeptide + Exocrocin (G2), and L1-E7 polypeptide + ExoCurcumin (G7) groups (*p* > 0.05). The groups immunized with L1-E7 polypeptide + ExoCrocin, and L1-E7 polypeptide + ExoCurcumin (G2 and G7) showed significantly higher levels of total IgG, IgG2a and IgG2b in comparison with L1-E7 polypeptide (G1; *p* < 0.05). Immunization with L1-E7 polypeptide + ExoCurcumin (G7) caused higher IgG2a antibody response than those with L1-E7 polypeptide + ExoCrocin (G2, *p* < 0.05). There is no antibody response against the recombinant polypeptide in control groups (**Fig 5**).

### Cytokine assay

**Fig 6:**
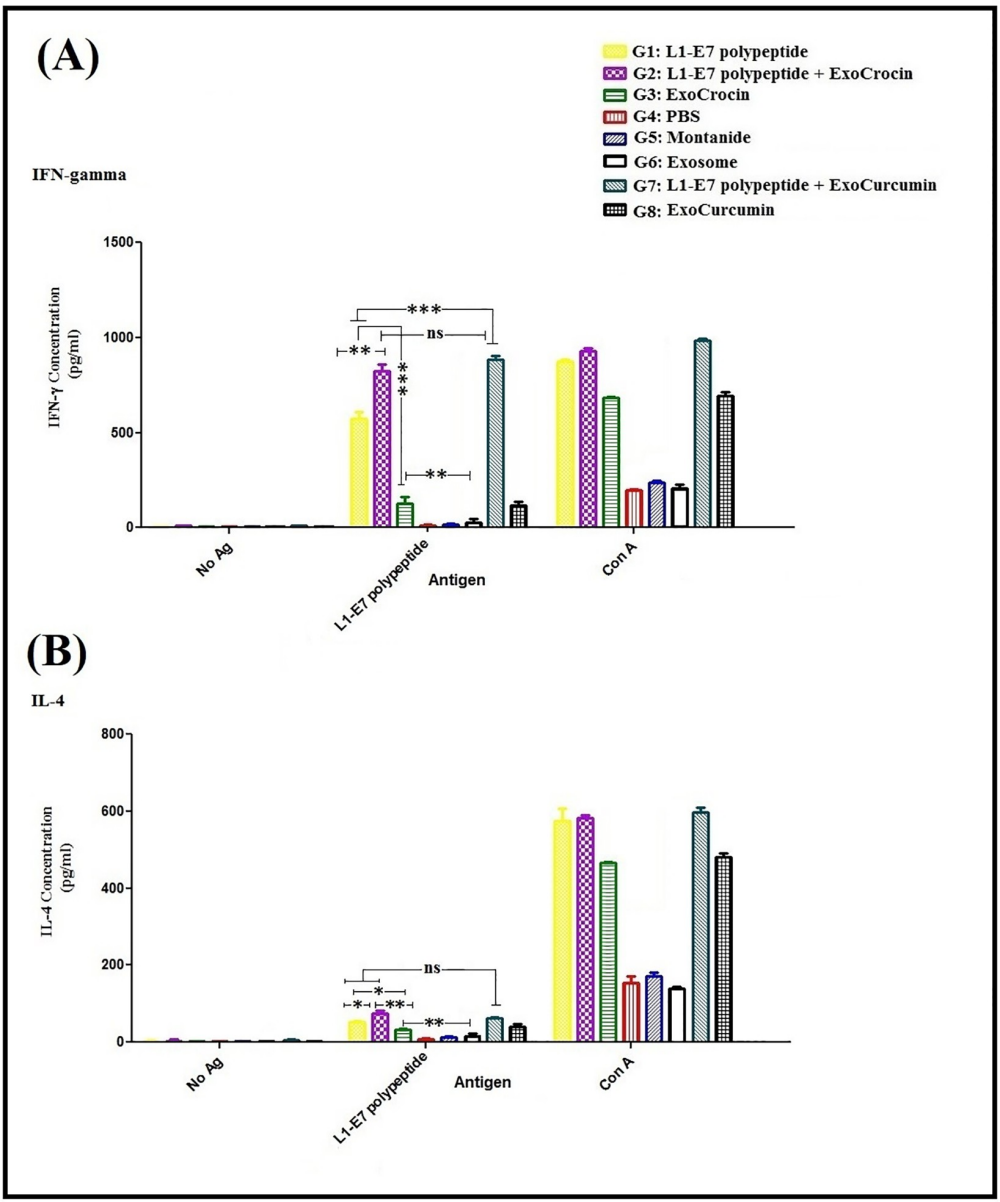
Evaluation of IFN-γ (A) and IL-4 (B) secretion in each group using sandwich ELISA: All analyses were performed in duplicate for each sample. The results were shown as mean absorbance at 450 nm ± SD. ns: non-significant; * *p* < 0.05; ** *p* < 0.01; *** *p* < 0.001.

**Fig 7:**
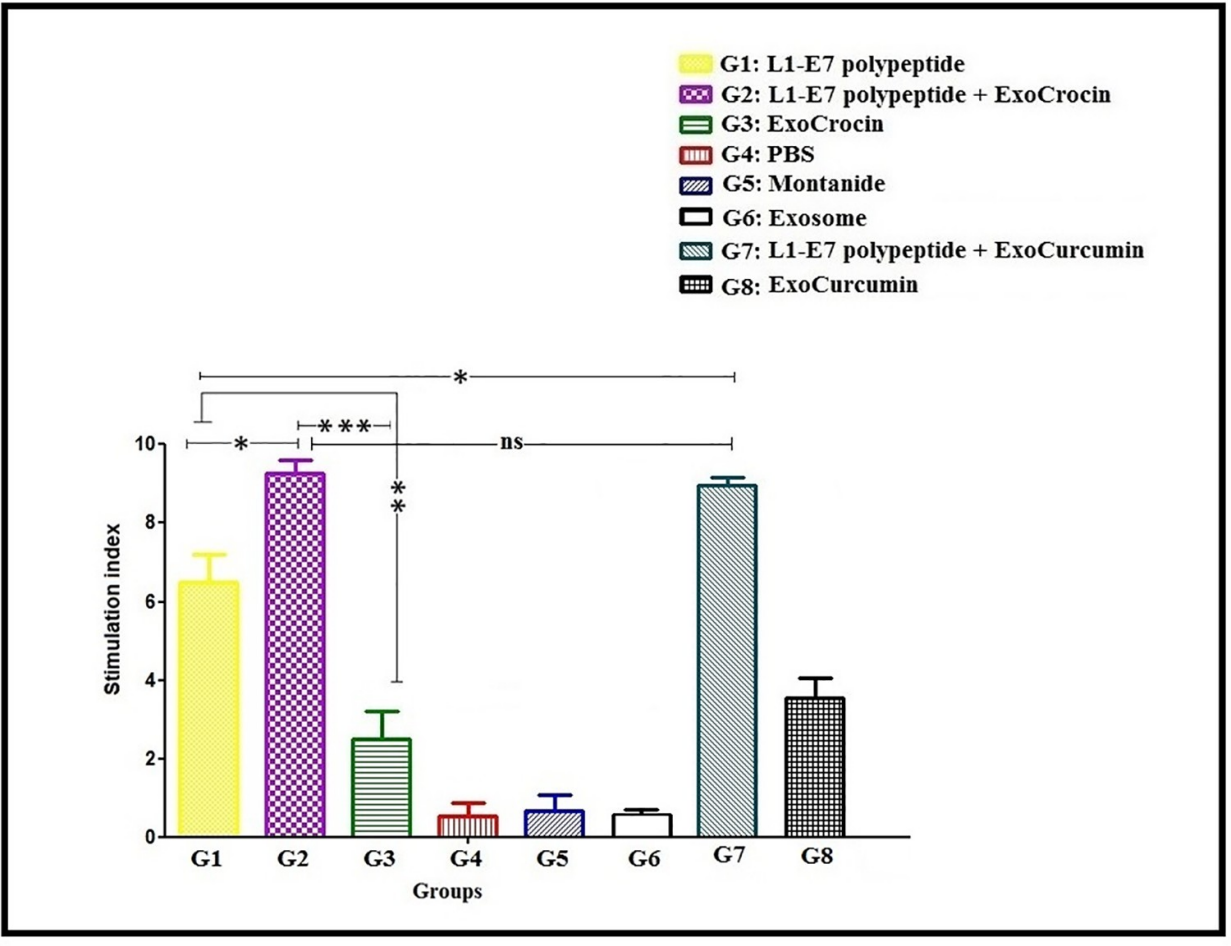
The lymphocyte proliferation assay in immunized groups with different regimens using MTT assay; ns: Non-significant; * *p* < 0.05; ** *p* < 0.01; *** *p* < 0.001.

The levels of IFN-γ and IL-4 cytokines were evaluated by ELISA determining Th1 and Th2 immune responses in mice, respectively. The groups injected with L1-E7 polypeptide, L1-E7 polypeptide + ExoCrocin, ExoCrocin, L1-E7 polypeptide + ExoCurcumin, and ExoCurcumin significantly increased the secretion of IFN-γ and IL-4 compared to the control groups (PBS, Montanide and Exosome; *p* < 0.01; **Fig 6**). The groups immunized with L1-E7 polypeptide + ExoCrocin (G2) and L1-E7 polypeptide + ExoCurcumin (G7) showed significantly higher levels of IFN-γ in comparison with ExoCrocin (G3) and ExoCurcumin groups (G8; *p* < 0.001). The levels of IFN-γ in groups injected with L1-E7 polypeptide + ExoCrocin (G2) and L1-E7 polypeptide + ExoCurcumin (G7) was higher than group injected with L1-E7 polypeptide (G1; *p* < 0.01). No significant difference in IFN-γ secretion was observed between L1-E7 polypeptide + ExoCrocin (G2) and L1-E7 polypeptide + ExoCurcumin (G7) groups (*p* > 0.05). The group immunized with L1-E7 polypeptide + ExoCrocin (G2) showed higher level of IL-4 than L1-E7 polypeptide (G1) and Exocrocin (G3; *p* < 0.05). However, the level of IL-4 secretion was significantly lower than IFN-γ in groups immunized with L1-E7 polypeptide (G1), L1-E7 polypeptide + ExoCrocin (G2), L1-E7 polypeptide + ExoCurcumin (G7). The ratio of IFN-γ to IL-4 was increased about 10–11 folds in groups immunized with L1-E7 polypeptide (G1), L1-E7 polypeptide + ExoCrocin (G2) and L1-E7 polypeptide + ExoCurcumin (G7) indicating direction of immune responses toward Th1 response. Moreover, ExoCrocin and ExoCurcumin could also induce cytokine secretion as compared to control groups (*p* < 0.01; **Fig 6**). The high rate of splenocyte proliferation was observed in groups receiving L1-E7 polypeptide + ExoCrocin (G2) and L1-E7 polypeptide + ExoCurcumin (G7; *p* < 0.05). The group injected with L1-E7 polypeptide (G1) showed higher level of lymphoproliferation in comparison with ExoCrocin (G3) and ExoCurcumin (G8) groups (*p* < 0.01). Our results indicated that the combination of exosomes carrying crocin or curcumin and L1-E7 polypeptide led to better Th-cell proliferative responses (**Fig 7**).

### Secretion of granzyme B

**Fig 8:**
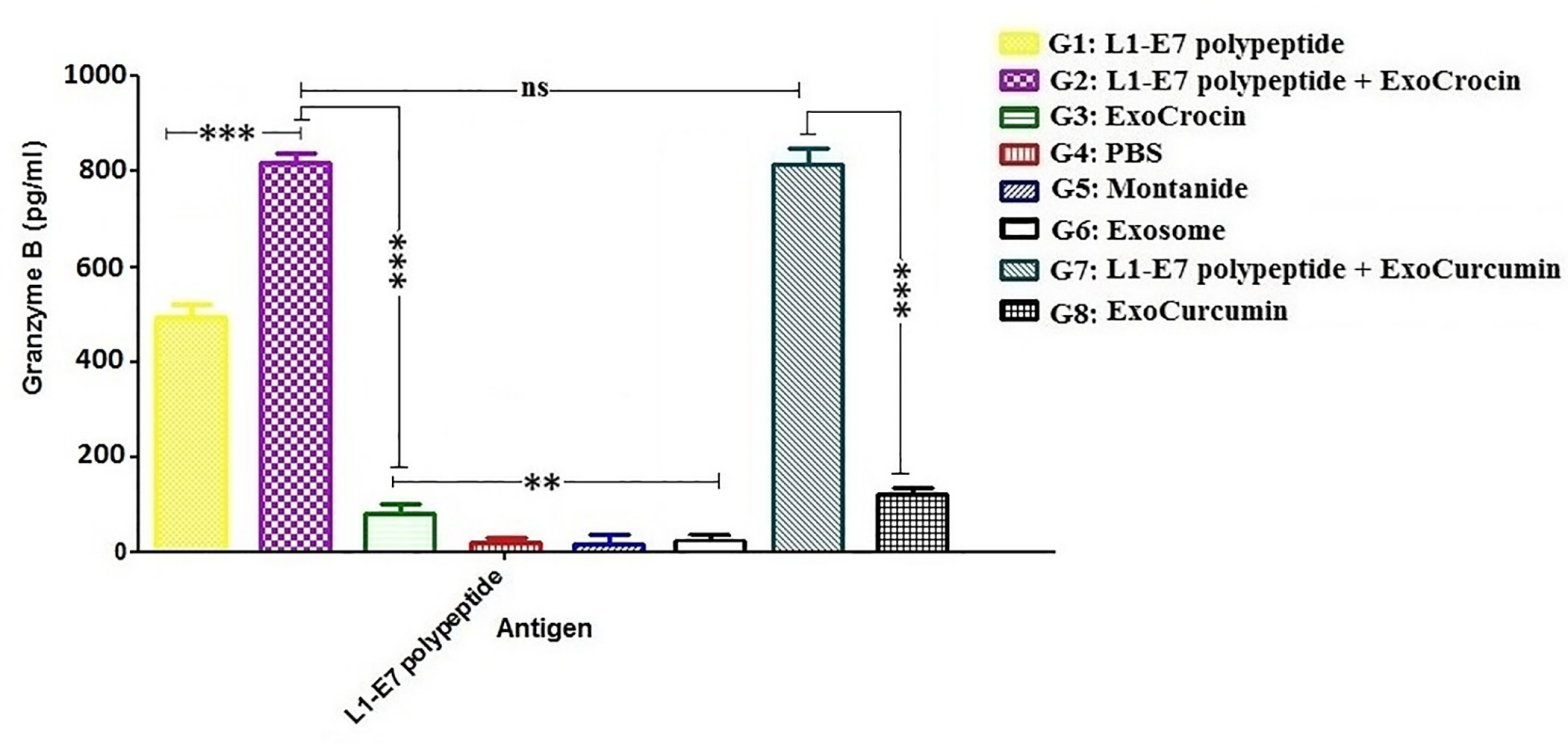
Evaluation of granzyme B secretion using ELISA. All analyses were performed in triplicate for each sample; ns: non-significant; * *p* < 0.05; ** *p* < 0.01; *** *p* < 0.001.

The secretion of Granzyme B in groups immunized with L1-E7 polypeptide (G1), L1-E7 polypeptide + ExoCrocin (G2) and L1-E7 polypeptide + ExoCurcumin (G7) was significantly higher than other groups (*p* < 0.001; **Fig 8**). The groups immunized with L1-E7 polypeptide + ExoCrocin (G2) and L1-E7 polypeptide + ExoCurcumin (G7) produced significantly higher concentrations of Granzyme B than group immunized with L1-E7 polypeptide (G1; *p* < 0.001). The level of Granzyme B in group receiving L1-E7 polypeptide + ExoCrocin (G2) was similar to that in group receiving L1-E7 polypeptide + ExoCurcumin (G7) (*p* > 0.05).

### Inhibition of tumor growth in mice

**Fig 9:**
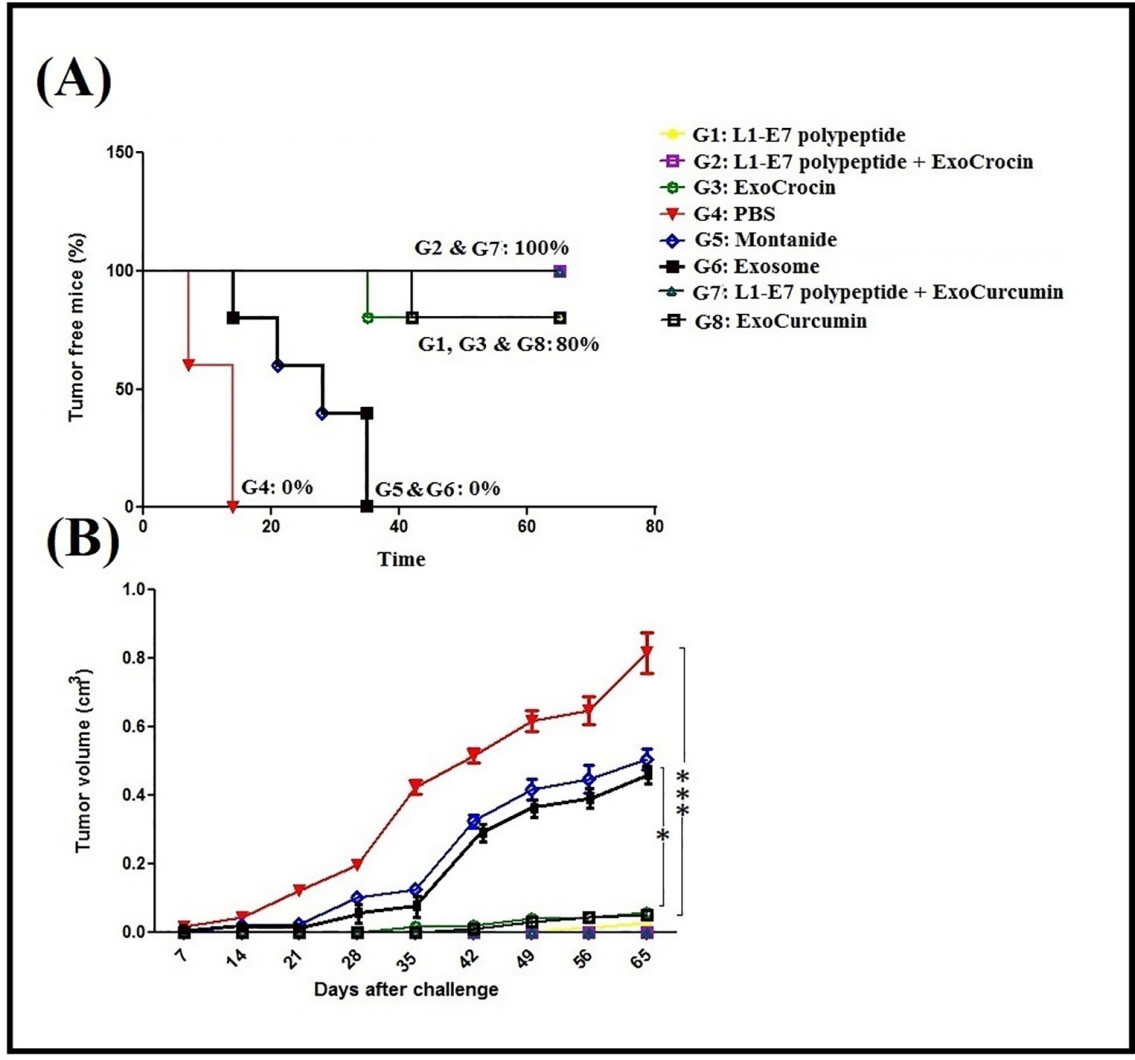
Prevention of tumor growth and survival percentage in immunized groups: (A) The percentage of tumor-free mice in immunized groups; (B) The measurement of tumor volume twice a week; ns: non-significant; * *p* < 0.05; ** *p* < 0.01; *** *p* < 0.001.

The prophylactic effects of different regimens were evaluated on tumor growth and survival rate. All groups were monitored after inoculation with TC-1 tumor cells for 65 days. The groups immunized with L1-E7 polypeptide + ExoCrocin (G2), and L1-E7 polypeptide + ExoCurcumin (G7) showed no tumor growth compared to other groups (*p* < 0.001). Indeed, these groups indicated 100% survival 65 days post-inoculation. The groups immunized with L1-E7 polypeptide (G1), ExoCrocin (G3) and ExoCurcumin (G8) showed significant reduction in tumor growth and 80% of them survived (**Fig 9**). Our results demonstrated that the control groups had tumor growth at 7–14 days. Generally, the simultaneous use of the recombinant L1-E7 polypeptide and exosomes harboring crocin or curcumin (G2 and G7) could protect 100% mice from tumor growth.

### Therapeutic effects

Mice with the established tumors (~4–5 mm^3^) were treated by the L1-E7 polypeptide + ExoCrocin (G1) and L1-E7 polypeptide + ExoCurcumin (G2). Both groups were free of tumor growth as compared to control group (PBS, G3; 100%).

## Discussion

Despite effective prophylactic vaccines and screening modalities for cervical dysplasia/cancer, it continues to be the fourth most common cancer in women worldwide with over 500,000 new cases every year [36]. Thus, design of an efficient therapeutic vaccine is necessary. Recently, multiepitope vaccines have the potential to induce responses restricted by a wide variety of HLA molecules and generate a balanced CD4^+^ and CD8^+^ T cells immunity [37]. In this study, our aim was to use a multiepitope vaccine construct (as an immunotherapy method) in combination with ExoCrocin or ExoCurcumin (as a chemotherapeutic agent) for improving immune responses and reducing tumor growth. In our previous study [27], the immunogenic and conserved L1 and E7 epitopes were selected to interact with human HLAs. Herein, their interaction with mice MHC alleles (*e*.*g*., C57BL/6 mice) was proved. Moreover, the lowest energy levels were observed for interaction of the L1-E7 multiepitope construct with TLRs indicating the highest binding affinity of the construct. Then, the L1-E7 multiepitope peptide construct was reversely translated to the multiepitope DNA construct. The DNA construct was expressed in bacterial system. The purified L1-E7 polypeptide was generated (~ 24 kDa), and its concentration was determined to use in immunological assay. The results of mice immunization indicated that the L1-E7 polypeptide stimulated B- and T-cell immune responses as compared to control groups. Our previous *in silico* study [27] showed the induction of IL-4 and IFN-γ cytokines by these selected epitopes. Indeed, all the selected epitopes (12 epitopes) had positive scores as IFN-γ inducers using the Hybrid-based (SVM + Motif) approach. Also, 11 epitopes showed the positive scores for IL-4 induction [27]. Herein, *in vivo* study showed that the L1-E7 polypeptide induced both IFN-γ and IL-4 cytokines in mice. Moreover, the level of IFN-γ secretion was significantly higher than IL-4 secretion.

Many studies focused on design of the peptide-based HPV vaccines using bioinformatics tools. Hosseini *et al*. predicted the immunogenic epitopes of L1 and L2 proteins in five types of HPV (11, 16, 18, 31 and 45) [38]. For L1 protein, the selected epitopes were NSGGYGGNPGQDNRVNVGMD, GTQCSNTSVQNGDCPPLELI (HPV-11), LDDTENASAYAANAGVDNRE, STSETTYKNTNFKEYLRHGE (HPV-16), ASTQSPVPGQYDATKFKQYS, NSSILEDWNFGVPPPPTTSL (HPV-18), HPYYSIPKSDNPKKIVVPKV, KGSPCSNNAITPGDCPPLEL (HPV-31), ASTQNPVPNTYDPTKFKHYS and NSSILENWNFGVPPPPTTSL (HPV-45) [38]. We studied 13 other types of HPVs (6, 33, 35, 39, 51, 52, 56, 58, 59, 68, 73, 82 & 83) as well as these five types for selection of L1 epitopes. Two conserved and immunogenic L1 epitopes were determined among 18 HPV types (**S1 Fig**). Namvar *et al*. designed DNA constructs based on the highly conserved and potentially immunogenic L1 and L2 epitopes in 13 types of high-risk HPVs (*i*.*e*., 16, 18, 31, 33, 35, 39, 45, 51, 52, 56, 58, 59 & 68). They reported that the combination of L1 and L2 DNA constructs without adjuvant or carrier induced effective immune responses *in vivo* [39]. In our study, the conserved and immunogenic L1 epitopes of 18 HPV types and E7 epitopes of five high-risk HPV types were selected and designed as a fusion DNA construct for generation of a recombinant L1-E7 polypeptide. Yazdani *et al*. selected the peptide sequences YVARTNIYYHAGTSRLLAVGHPYFPIK (MHC-I: A0101, A2601, B3901, A2902, B3501 and MHC-II: DR15 (DRB1*1501), DR4, DR11 (DRB1*1101), DR1 (DRB1*0101), DR4 (DRB1*0401), DR51 (DRB5*0101)) and YGDSLFFYLRREQMFVRH (MHC-I: B0702, C1402 and MHC-II: DR51 (DRB5*0101)) using immunoinformatics tools to design an L1-based peptide vaccine [40]. Jabbar *et al*. predicted QAEPDRAHY and AEPQRHTML peptides as antigenic E7 peptides in HPV types 16 and 18, respectively [41]. In our study, the immunogenic E7 epitopes of other high-risk HPV types (16, 18, 31, 33 & 45) were determined by considering various factors, as well. In this regard, Panahi *et al*. identified the immunogenic CD8^+^ T-cell epitopes in HPV 16, 18, 31 and 45 using a two-step method consisting of molecular docking and sequence-based approaches [42]. They suggested RAHYNIVTF, STHVDIRTL, LEDLLMGTL, LQPETTDLY & TLHEYMLDL epitopes for HPV16 E7, SSADDLRAF, FQQLFLNTL, QLFLNTLSF, TLQDIVLHL & LRAFQQLFL epitopes for HPV18 E7, TSNYNIVTF, TLQDYVLDL, GQAEPDTSNY, QAEPDTSNY & QPEATDLHCY epitopes for HPV31 E7, TLQEIVLHL, NELDPVDLL, LQQLFLSTL, QLFLSTLSF & SSAEDLRTL epitopes for HPV45 E7 [42]. Our epitopes contain these epitopes with longer lengths as well as HPV33 E7 epitopes (**S1 Fig**).

The extracellular vesicles including exosomes were known as potential drug carriers. Two common methods for isolation of exosomes are the use of commercial kits and ultracentrifuge. Based on the results presented by Skottvoll *et al*., the quality of ultracentrifuge/ commercial kit isolation methods were approximately equal. Moreover, kit isolation needs less starting material than conventional ultracentrifuge equipment [43]. In this study, ExoQuick-TC ULTRA kit was used to isolate exosomes from human embryonic kidney (HEK)-293T cells [44]. Extracellular vesicles (EVs) are secreted by all human tissues, and isolated from available cell lines such as HEK-293T cells. HEK-293T cell line is a suitable source due to the lack of cancer-related and immunogenic-induction mechanisms, thus representing a safe source of extracellular vesicles under *in vitro* and *in vivo* conditions [45]. The ability of exosomes was proved to deliver efficiently in solid tumors [46]. Our study showed that the exosomes extracted from HEK-293T cells can be transferred to tumor cells *in vitro*.

Exosomes could increase the therapeutic index of drugs. Hadla and coworkers showed that doxorubicin loaded in exosomes was safer and more effective than free doxorubicin. Indeed, higher doses of the drug can be delivered by the exosome [47]. Li *et al*. showed that injection of ExoGEM (Exosome loaded with Gemcitabine) led to significant reduction of tumor growth after treatment. ExoGEM could enhance survival rate in tumor-challenged mice in a dose-dependent manner [22]. On the other hand, many phytochemicals could considerably inhibit the growth of tumor cells by inducing cell cycle arrest or apoptosis. Wang *et al*. investigated the effects of various concentrations of curcumin (0, 5, 15, 30 & 50 μM) on the proliferation and morphology of SiHa cells (a cervical carcinoma cell line). The percentage of cell proliferation was considerably decreased at concentration of 50 μM in a dose-dependent manner [48]. The studies showed that free curcumin is highly hydrophobic and cannot be administered systemically. Therefore, liposome/ exosome-based delivery methods were used efficiently to encapsulate free curcumin which resolve the problem of poor oral availability, and intravenous dosing [49–51]. Sun *et al*. showed that intraperitoneal injection of curcumin loaded in exosome (exosomal curcumin) led to a five- to ten-fold higher curcumin accumulated in peripheral blood than that of curcumin alone. At 12 hours after intraperitoneal injection, curcumin in the plasma still remained at a high level in mice injected with exosomal curcumin [50].

Curcumin reduces the side effects of chemotherapy or radiotherapy resulting in improving patients’ quality of life. The findings from several studies suggest that curcumin can prevent the formation and spread of tumors or reduce their size by exerting anti-angiogenic effects, inducing apoptosis and interfering with the cell proliferation cycle. Curcumin inhibits angiogenesis in some tumors by suppressing angiogenic cytokines such as IL-6, IL-23, and IL-1β [52].

The cytotoxic effects of saffron and its components were studied in human tumor cells. *In vitro* cytotoxic assay showed that saffron extract inhibited the growth of tumor cells, whereas, non-tumor cells were less sensitive or even insensitive to the extract. Escribano *et al*. indicated that crocin induced apoptosis in HeLa cells (a cervical carcinoma cell line). Moreover, human fibroblasts were less sensitive to saffron extract than HeLa cells, suggesting a selective cytotoxicity on tumor cells [53]. In 2018, Jiang *et al*. investigated the anticancer activity of crocin against HeLa cells. Cell viability was reduced 97.1, 96.4, 85.5, 78.4 and 70.2% at 25, 50, 75, 100 and 125 mg/L of crocin, respectively. These data suggested that crocin is a potential antitumor agent against cervical carcinoma [54]. In another study, HeLa cells were incubated with crocin (1, 2 & 4 mM), and liposomal crocin (0.5 & 1 mM). Liposomal crocin showed higher cytotoxic effects than free crocin in HeLa cells. Liposomal encapsulation enhanced apoptogenic effects of crocin on tumor cells [55]. Our study showed that crocin and curcumin could be loaded efficiently in exosomes isolated from HEK-293T cells, and the exosomal forms of curcumin or crocin were significantly non-toxic to non-tumor HEK-293T cells.

In our study, the L1-E7 polypeptide construct along with exosomes loaded with crocin or curcumin (ExoCrocin or ExoCurcumin) was injected in mice. The health of mice after injection and the lack of inflammation represented that the tested doses of L1-E7 polypeptide, ExoCurcumin and ExoCrocin had no side effects in mice, as shown in MTT assay. Our finding indicated that combination of ExoCurcumin or ExoCrocin with L1-E7 polypeptide (G2 & G7) could significantly enhance the levels of total IgG, IgG2a, IgG2b, IFN-γ, Granzyme B and lymphocyte proliferation as compared to L1-E7 polypeptide (G1), ExoCrocin (G3), ExoCurcumin (G8) and control groups (G4-G6). Moreover, the levels of IgG1 and IL-4 were increased in groups immunized with L1-E7 polypeptide (G1), L1-E7 polypeptide/ ExoCrocin (G2), and L1-E7 polypeptide/ ExoCurcumin (G7) as compared to ExoCrocin (G3), ExoCurcumin (G8) and control groups (G4-G6). However, the ratios of IFN-γ: IL-4, and IgG2a: IgG1 in groups immunized with L1-E7 polypeptide/ ExoCrocin (G2), L1-E7 polypeptide/ ExoCurcumin (G7) and then L1-E7 polypeptide (G1) were significantly higher than other groups indicating induction of immune responses toward Th1 responses. Indeed, the combination of ExoCurcumin or ExoCrocin with a polypeptide improved cellular immune responses toward Th1 response, and CTL activity. In addition, groups injected with L1-E7 polypeptide combined with ExoCrocin (G2) or ExoCurcumin (G7) elicited the same immune responses indicating similar effects of ExoCrocin and ExoCurcumin at a certain non-toxic concentration. Protective studies showed that L1-E7 polypeptide + ExoCrocin (G2), and L1-E7 polypeptide + ExoCurcumin (G7) could confer complete protection against TC-1 tumor-challenged mice (survival rate: ~ 100%) as compared to L1-E7 polypeptide (G1), ExoCrocin (G3) and ExoCurcumin (G8) (survival rate: ~ 80%). The results indicated that injection of exosomes loaded with crocin or curcumin along with L1-E7 polypeptide has a significant therapeutic effect on tumor eradication, as well. On the other hand, ExoCrocin and ExoCurcumin induced total IgG, IgG1, IgG2a, IgG2b, IFN-γ and IL-4 as compared to control groups indicating the possible immunological response of exosomal compounds, because the empty exosomes extracted from HEK-293T cells induced no considerable immune responses. For instance, in 2018, Zhang *et al*. indicated that crocin significantly promoted T cell proliferation and the secretion of IL-2 and IL-4 in a dose-dependent manner [56]. It was reported that the exosomes are non-immunogenic in nature due to similar composition as body’s own cells [57]. Vega *et al*. also showed the role of Hsp70-positive exosomes in the activation of macrophages by assessment of the TNF-α secretion which was about 260-fold higher than the recombinant Hsp70 [58].

There are various reports about the efficiency of dendritic cells/ tumor cells-derived exosomes *in vitro* and *in vivo* experiments. For example, in 2020, Hong *et al*. indicated that dendritic cell (DC)-derived engineered antigenic exosomes (presenting peptides from the M, NS and L proteins of respiratory syncytial virus) had the potency to induce antigen-specific CD8^+^ T cell activation *in vitro* and *in vivo* [59]. However, the exosomes derived from DCs or tumor cells are immunogenic and may stimulate immune response without the use of antigen, as well. In this study, we used the exosomes derived from a non-immunogenic cell line (as a carrier) for reduction of side effects and evaluation of the loaded compounds.

## Conclusion

In conclusion, our data showed that combination of ExoCurcumin or ExoCrocin with L1-E7 polypeptide (G2 & G7) could significantly enhance the levels of total IgG, IgG2a, IgG2b, IFN-γ, Granzyme B and lymphocyte proliferation indicating induction of Th1 response and CTL activity. The protective and antitumor effects of these regimens confirmed the induced cellular immune responses *in vivo*. It was interesting that ExoCurcumin or ExoCrocin showed similar immunological and antitumor effects at a certain non-toxic dose. However, this study is the first report for construction of a novel fusion L1-E7 polypeptide, preparation of Exosomal crocin and curcumin, immunological assay of the L1-E7 multiepitope construct with and without Exosomal crocin and curcumin, and their protective and therapeutic effects in tumor mice. However, the study of cellular uptake mechanisms of the exosomal crocin and curcumin forms require further researches in the next studies.

## Supporting information

S1 Fig Molecular docking between the CTL epitopes and mouse MHC class I alleles: The successful docking between L1 epitope (DLDQFPLGRKFLLQ) and H-2-Db with interaction score of 263.0 (A); between E7 epitope (HGPKATVQDIVLHL) and H-2-Ld with interaction score of 352.0 (B); between E7 (AEPDRAHYNIVTF) and H-2-Kb with interaction score of 226.0 (C); between E7 epitope (RPDGQAQPATADYYI) and H-2-Kd with interaction score of 267.0 (D); The mouse MHC alleles were shown as golden ribbon representation, and the CTL epitopes were shown as colored ball & stick representation.
(TIF)

S2 Fig Molecular docking between the L1-E7 multiepitope construct and TLRs. A) Interaction of the multiepitope construct with TLR-2 (A), TLR-3 (B), TLR-4 (C), TLR-5 (D), TLR-8 (E), and TLR-9 (F). The multiepitope construct was shown as colored ribbon representation, and TLRs were indicated as golden ribbon presentation.
(TIF)

S3 Fig Expression and purification of GST-L1-E7 protein in *E*. *coli* system. A) SDS-PAGE analysis: lane 1: Molecular weight (10–140 kDa, Fermentas), lane 2: Before induction, lane 3: 3 h after induction, and lane 4: Purified protein; B) Identification of the recombinant GST-L1-E7 protein by western blot analysis using anti-His antibody: lane 1: Molecular weight, lane 2: Before induction, lane 3: After induction, and lane 4: Purified protein.
(TIF)

S4 Fig Characterization of HEK-293T-derived exosomes. A) Scanning electron microscopy (SEM; 100000× magnification); B) Transmission electron microscopy (TEM). The vesicle shape and membrane integrity of exosomes were proved in these images.
(TIF)

S1 Table The selected peptides for HPV L1/ E7. (DOCX)

S2 Table CTL epitope prediction for mouse MHC class I alleles. (DOCX)

S3 Table HTL epitope prediction for mouse MHC class II alleles. (DOCX)

S4 Table Peptide-MHC interaction similarity scores between the CTL epitopes and mouse MHC class I alleles. (DOCX)

S5 Table Absorbance of crocin and curcumin in different conditions. (DOCX)
