# Supplementary figures and images for: Synergistic effects of exosomal crocin or curcumin compounds and HPV L1-E7 polypeptide vaccine construct on tumor eradication in C57BL/6 mouse model
Source: PLoS One. 2021 Oct 14;16(10):e0258599. doi: 10.1371/journal.pone.0258599 (PMC8516259; doi:10.1371/journal.pone.0258599)

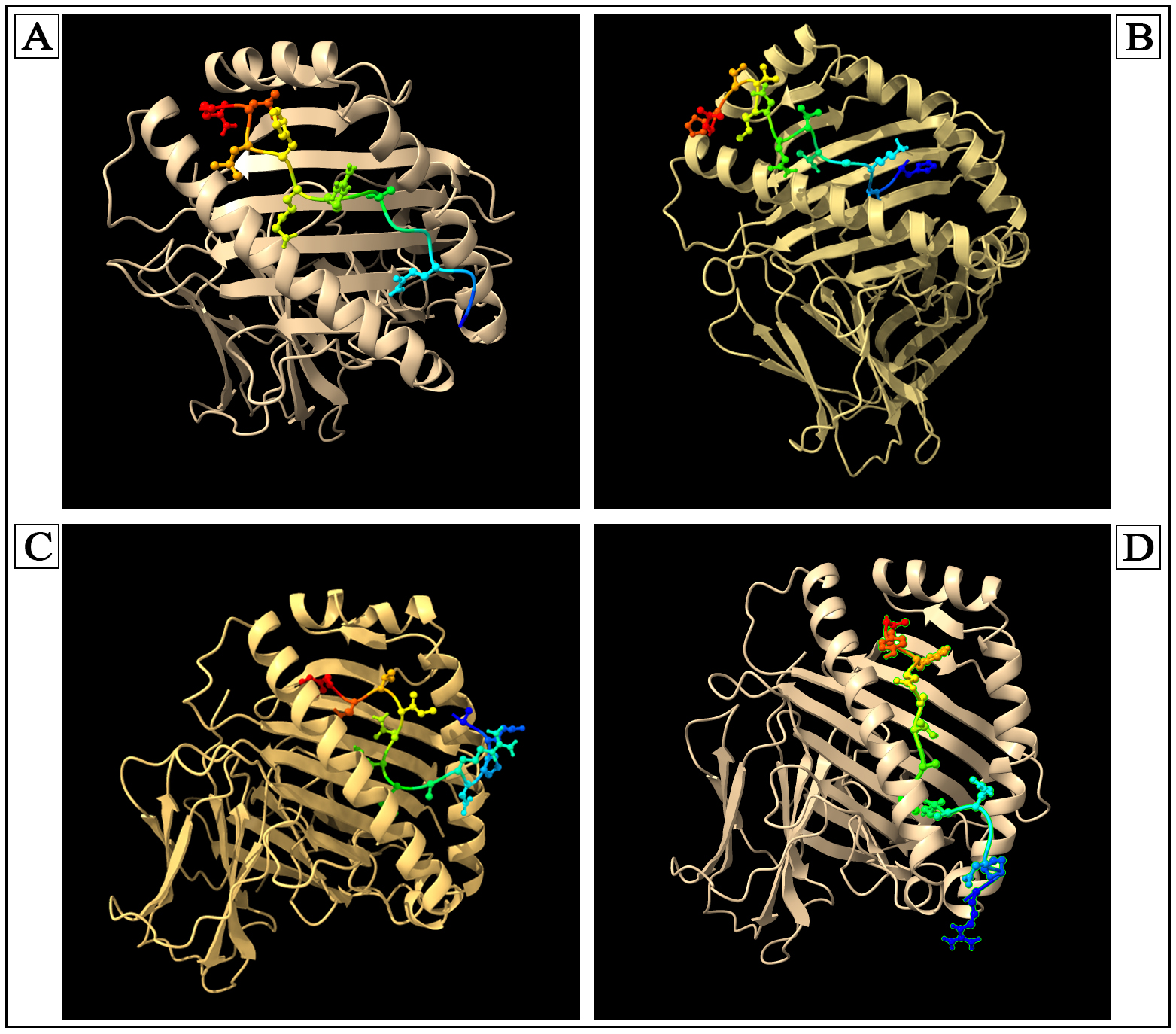

Supplement: S1 Fig — Molecular docking between the CTL epitopes and mouse MHC class I alleles: The successful docking between L1 epitope (DLDQFPLGRKFLLQ) and H-2-Db with interaction score of 263.0 (A); between E7 epitope (HGPKATVQDIVLHL) and H-2-Ld with interaction score of 352.0 (B); between E7 (AEPDRAHYNIVTF) and H-2-Kb with interaction score of 226.0 (C); between E7 epitope (RPDGQAQPATADYYI) and H-2-Kd with interaction score of 267.0 (D); The mouse MHC alleles were shown as golden ribbon representation, and the CTL epitopes were shown as colored ball & stick representation. (TIF) [file pone.0258599.s001.tif]

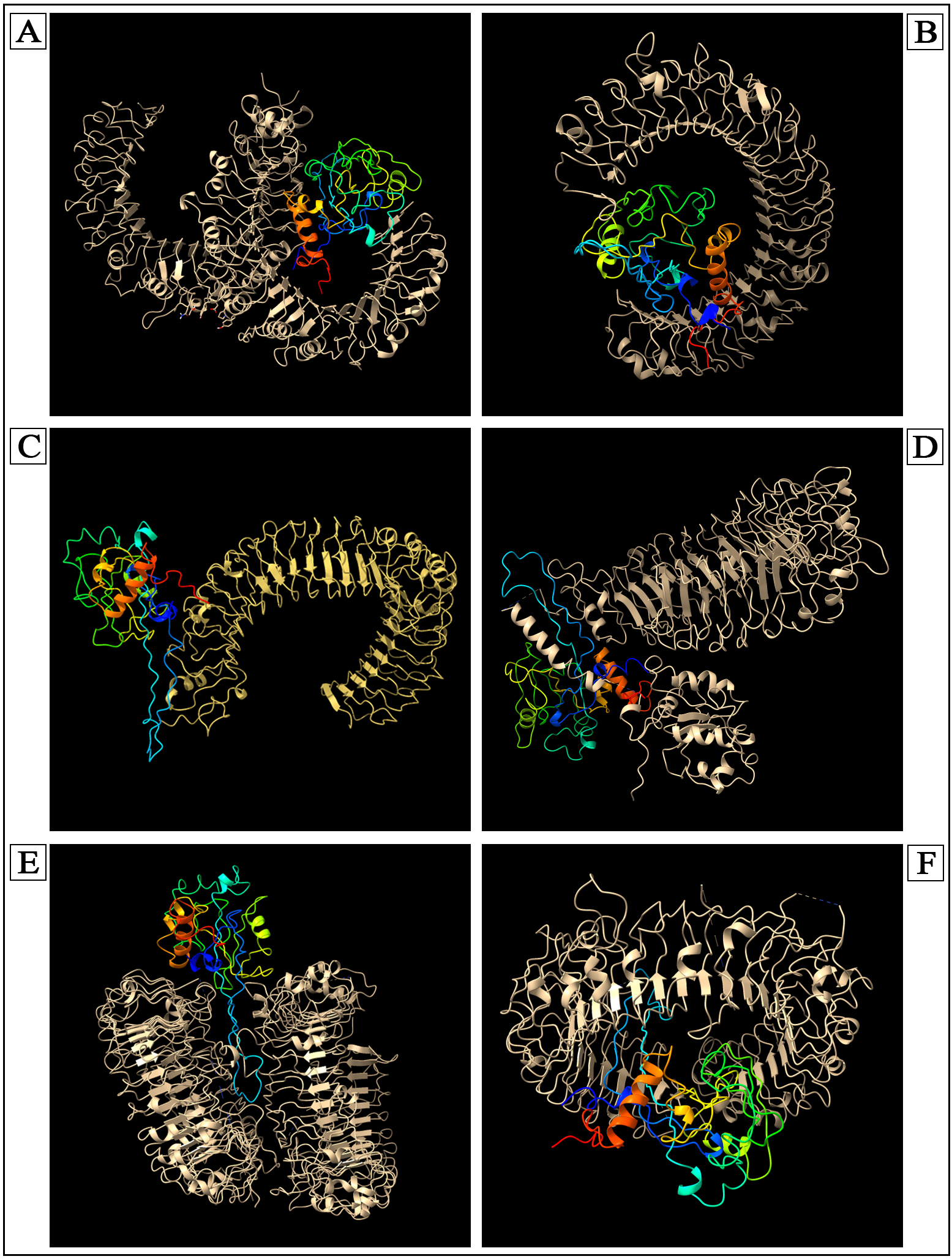

Supplement: S2 Fig — A) Interaction of the multiepitope construct with TLR-2 (A), TLR-3 (B), TLR-4 (C), TLR-5 (D), TLR-8 (E), and TLR-9 (F). The multiepitope construct was shown as colored ribbon representation, and TLRs were indicated as golden ribbon presentation. (TIF) [file pone.0258599.s002.tif]

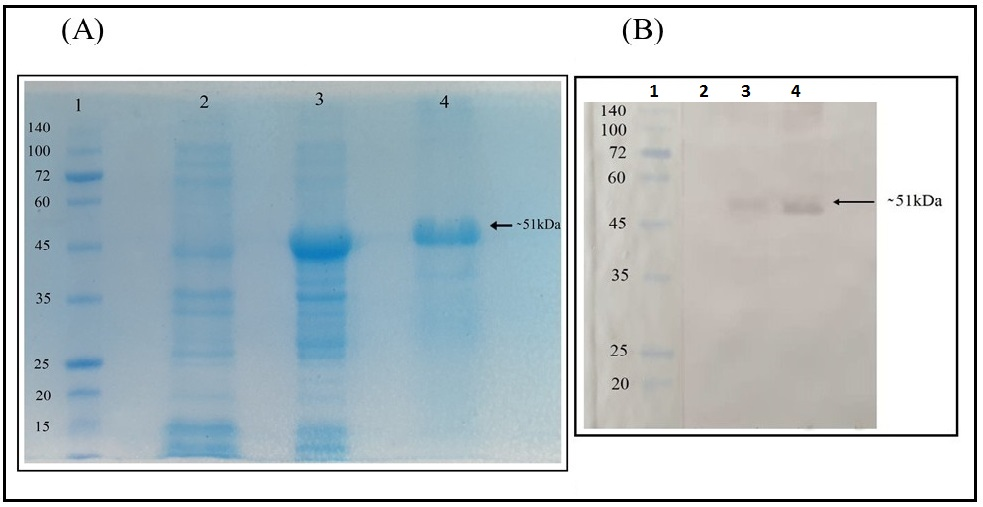

Supplement: S3 Fig — A) SDS-PAGE analysis: lane 1: Molecular weight (10–140 kDa, Fermentas), lane 2: Before induction, lane 3: 3 h after induction, and lane 4: Purified protein; B) Identification of the recombinant GST-L1-E7 protein by western blot analysis using anti-His antibody: lane 1: Molecular weight, lane 2: Before induction, lane 3: After induction, and lane 4: Purified protein. (TIF) [file pone.0258599.s003.tif]

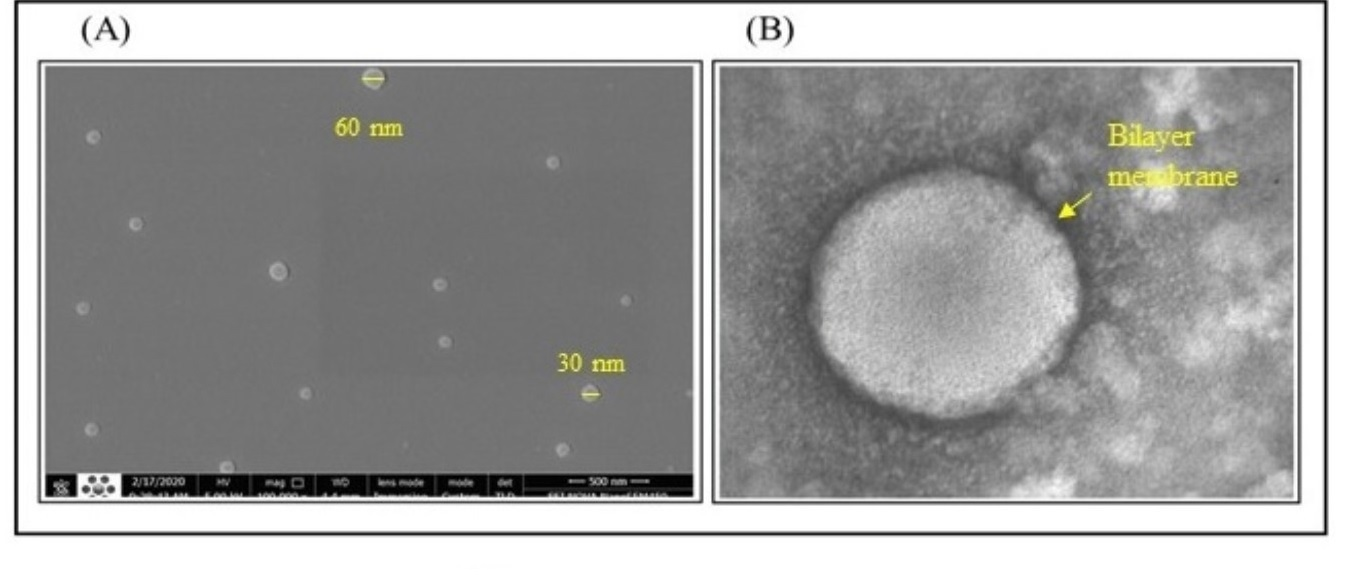

Supplement: S4 Fig — A) Scanning electron microscopy (SEM; 100000× magnification); B) Transmission electron microscopy (TEM). The vesicle shape and membrane integrity of exosomes were proved in these images. (TIF) [file pone.0258599.s004.tif]
