# Supplementary material for: Synergistic effects of exosomal crocin or curcumin compounds and HPV L1-E7 polypeptide vaccine construct on tumor eradication in C57BL/6 mouse model
Source: PLoS One. 2021 Oct 14;16(10):e0258599. doi: 10.1371/journal.pone.0258599 (PMC8516259; doi:10.1371/journal.pone.0258599)
Supplement: S1 Table — (DOCX) [file pone.0258599.s005.docx]

**Supplementary Table 1:** The selected peptides for HPV L1/ E7

| **Protein** | **HPV type** | **Position** | **Sequence (Match MHC)** |
| --- | --- | --- | --- |
| L1 | 18 types of HPV | 458-471  327-341 | DLDQFPLGRKFLLQ (MHC-I)  NQLFVTVVDTTRSTN (MHC-II) |
| E7 | Type 16 | 45-57  7-21 | AEPDRAHYNIVTF (MHC-I)  TLHEYMLDLQPETTD (MHC-II) |
| E7 | Type 18 | 2-15  83-97 | HGPKATVQDIVLHL (MHC-I)  LRAFQQLFLNTLSFV (MHC-II) |
| E7 | Type 31 | 46-57  6-20 | KPDTSNYNIVTF (MHC-I)  PTLQDYVLDLQPEAT (MHC-II) |
| E7 | Type 33 | 40-54  8-22 | RPDGQAQPATADYYI (MHC-I)  LKEYVLDLYPEPTDL (MHC-II) |
| E7 | Type 45 | 85-95  87-101 | RTLQQLFLSFV (MHC-I)  LQQLFLSTLSFVCPW (MHC-II) |
