# Supplementary material for: Synergistic effects of exosomal crocin or curcumin compounds and HPV L1-E7 polypeptide vaccine construct on tumor eradication in C57BL/6 mouse model
Source: PLoS One. 2021 Oct 14;16(10):e0258599. doi: 10.1371/journal.pone.0258599 (PMC8516259; doi:10.1371/journal.pone.0258599)
Supplement: S2 Table — (DOCX) [file pone.0258599.s006.docx]

**Supplementary Table 2:** CTL epitope prediction for mouse MHC class I alleles

| **Epitopes** | **Mouse allele** | **Percentile Rank (IEDB)** | **Mouse allele** | **NetMHCpan4.1** | **Mouse allele** | **SYFPEITHI** |
| --- | --- | --- | --- | --- | --- | --- |
| **L1 protein** |  |  |  |  |  |  |
| DLDQFPLGRKFLLQ | H-2-Kk | 0.31 | H-2-Ld | 0.069 | H-2-Db | 15 |
|  | H-2-Ld | 0.05 | H-2-Kk | 0.439 | H-2-Kb | 13 |
|  | H-2-Kd | 0.57 | H-2-Kd | 0.764 | H-2-Kd | 18 |
|  | H-2-Kb | 1.9 | H-2-Dd | 1.851 | H-2-Kk | 10 |
|  |  |  | H-2-Kb | 2.965 | H-2-Ld | 21 |
|  |  |  | H-2-Db | 4.907 |  |  |
| **E7 protein** |  |  |  |  |  |  |
| AEPDRAHYNIVTF | H-2-Db | 0.01 | H-2-Db | 0.011 | H-2-Db | 23 |
|  | H-2-Kk | 0.54 | H-2-Dd | 0.135 | H-2-Kb | 16 |
|  | H-2-Kb | 0.23 | H-2-Kb | 0.468 | H-2-Kd | 11 |
|  | H-2-Ld | 0.38 | H-2-Kd | 0.820 | H-2-Kk | 19 |
|  | H-2-Dd | 0.09 | H-2-Kk | 0.776 | H-2-Ld | 20 |
|  | H-2-Kd | 0.62 | H-2-Ld | 0.555 |  |  |
| HGPKATVQDIVLHL | H-2-Kb | 0.25 | H-2-Db | 1.147 | H-2-Db | 17 |
|  | H-2-Kk | 1.0 | H-2-Dd | 0.141 | H-2-Kb | 12 |
|  | H-2-Ld | 0.42 | H-2-Kb | 0.498 | H-2-Kd | 16 |
|  | H-2-Dd | 0.1 | H-2-Kd | 1.358 | H-2-Kk | 13 |
|  | H-2-Db | 0.84 | H-2-Kk | 1.360 | H-2-Ld | 17 |
|  | H-2-Kd | 0.96 | H-2-Ld | 0.608 |  |  |
| KPDTSNYNIVTF | H-2-Kb | 0.03 | H-2-Db | 0.048 | H-2-Db | 24 |
|  | H-2-Db | 0.04 | H-2-Dd | 0.289 | H-2-Kb | 16 |
|  | H-2-Ld | 0.15 | H-2-Kb | 0.077 | H-2-Kd | 16 |
|  | H-2-Kk | 1.6 | H-2-Kd | 3.748 | H-2-Kk | 19 |
|  | H-2-Dd | 0.2 | H-2-Kk | 2.199 | H-2-Ld | 20 |
|  | H-2-Kd | 2.7 | H-2-Ld | 0.262 |  |  |
| RPDGQAQPATADYYI | H-2-Ld | 0.35 | H-2-Db | 0.448 | H-2-Db | 19 |
|  | H-2-Db | 0.3 | H-2-Kk | 2.810 | H-2-Kb | 7 |
|  | H-2-Kk | 2.1 | H-2-Kd | 3.240 | H-2-Kd | 15 |
|  | H-2-Kd | 2.3 | H-2-Ld | 0.528 | H-2-Kk | 11 |
|  |  |  |  |  | H-2-Ld | 19 |
| RTLQQLFLSFV | H-2-Ld | 0.53 | H-2-Dd | 1.659 | H-2-Db | 13 |
|  | H-2-Kb | 0.93 | H-2-Kb | 1.593 | H-2-Kb | 11 |
|  | H-2-Kk | 3.6 | H-2-Ld | 0.780 | H-2-Kd | 13 |
|  | H-2-Dd | 1.2 | H-2-Kd | 4.750 | H-2-Kk | 11 |
|  | H-2-Db | 3.9 | H-2-Kk | 4.801 | H-2-Ld | 14 |
