# Supplementary material for: Synergistic effects of exosomal crocin or curcumin compounds and HPV L1-E7 polypeptide vaccine construct on tumor eradication in C57BL/6 mouse model
Source: PLoS One. 2021 Oct 14;16(10):e0258599. doi: 10.1371/journal.pone.0258599 (PMC8516259; doi:10.1371/journal.pone.0258599)
Supplement: S3 Table — (DOCX) [file pone.0258599.s007.docx]

**Supplementary Table 3:** HTL epitope prediction for mouse MHC class II alleles

| **Epitopes** | **Mouse allele** | **Percentile Rank (IEDB)** | **Mouse allele** | **SYFPEITHI** |
| --- | --- | --- | --- | --- |
| **L1 protein** |  |  |  |  |
| NQLFVTVVDTTRSTN | H2-IEd | 12.50 | H2-IAd | 11 |
|  | H2-IAd | 27.50 | H2-IEd | 18 |
|  |  |  | H2-IEk | 20 |
| **E7 protein (HPV type)** |  |  |  |  |
| TLHEYMLDLQPETTD | H2-IAb | 37.00 | H2-IAd | 17 |
|  | H2-IAd | 41.50 | H2-IEk | 6 |
| LRAFQQLFLNTLSFV | H2-IAb | 13.00 | H2-IAd | 21 |
|  | H2-IEd | 23.50 | H2-IEd | 12 |
| PTLQDYVLDLQPEAT | H2-IAb | 19.70 | H2-IAd | 10 |
|  |  |  | H2-IEk | 6 |
| LKEYVLDLYPEPTDL | H2-IEd | 29.50 | H2-IEd | 10 |
|  | H2-IAb | 30.00 | H2-IEk | 6 |
| LQQLFLSTLSFVCPW | H2-IEd | 20.00 | H2-IAd | 14 |
|  | H2-IAb | 22.90 | H2-IEd | 8 |
|  | H2-IAd | 27.50 | H2-IEk | 16 |
