# Supplementary material for: Synergistic effects of exosomal crocin or curcumin compounds and HPV L1-E7 polypeptide vaccine construct on tumor eradication in C57BL/6 mouse model
Source: PLoS One. 2021 Oct 14;16(10):e0258599. doi: 10.1371/journal.pone.0258599 (PMC8516259; doi:10.1371/journal.pone.0258599)
Supplement: S4 Table — (DOCX) [file pone.0258599.s008.docx]

**Supplementary Table 4:** Peptide-MHC interaction similarity scores between the CTL epitopes and mouse MHC class I alleles

| **Epitopes** | **H2-Db** | **H2-Dd** | **H-2-Kb** | **H-2-Kd** | **H-2-Ld** |
| --- | --- | --- | --- | --- | --- |
| **L1 protein** |  |  |  |  |  |
| DLDQFPLGRKFLLQ | 263.0 | 224.0 | 235.0 | 254.0 | 324.0 |
| **E7 protein (HPV type)** |  |  |  |  |  |
| AEPDRAHYNIVTF | 243.0 | 224.0 | 226.0 | 239.0 | 330.0 |
| HGPKATVQDIVLHL | 254.0 | 241.0 | 212.0 | 260.0 | 352.0 |
| KPDTSNYNIVTF | 226.0 | 181.0 | 203.0 | 232.0 | 322.0 |
| RPDGQAQPATADYYI | 259.0 | 218.0 | 194.0 | 267.0 | 358.0 |
| RTLQQLFLSFV | 269.0 | 202.0 | 187.0 | 238.0 | 281.0 |
