# Supplementary material for: Synergistic effects of exosomal crocin or curcumin compounds and HPV L1-E7 polypeptide vaccine construct on tumor eradication in C57BL/6 mouse model
Source: PLoS One. 2021 Oct 14;16(10):e0258599. doi: 10.1371/journal.pone.0258599 (PMC8516259; doi:10.1371/journal.pone.0258599)
Supplement: S5 Table — (DOCX) [file pone.0258599.s009.docx]

**S5 Table.** Absorbance of crocin and curcumin in different conditions

|  | Absorption of the initial crocin solution | Absorption of crocin+ exosomes (Sonicate) | Absorption of crocin+ exosomes (Freeze-thaw) | Absorption of crocin+ exosomes+RIPA (Sonicate) | Absorption of crocin+ exosomes+RIPA (Freeze-thaw) |
| --- | --- | --- | --- | --- | --- |
| OD  (430-450 nm) | 2.93 | 1.86 | 1.09 | 2.02 | 2.73 |
|  | Absorption of the initial curcumin solution | Absorption of curcumin+ exosomes (Sonicate) | Absorption of curcumin+ exosomes (Freeze-thaw) | Absorption of curcumin+ exosomes+RIPA (Sonicate) | Absorption of curcumin+ exosomes+RIPA (Freeze-thaw) |
| OD  (410-430 nm) | 1.85 | 0.9 | 0.4 | 1.78 | 1.8 |
